# Development and evaluation of a facile mesh-to-surface tool for customised wheelchair cushions

**DOI:** 10.1186/s41205-022-00165-5

**Published:** 2023-02-13

**Authors:** Susan Nace, John Tiernan, Aisling Ní Annaidh, Donal Holland

**Affiliations:** 1 School of Mechanical and Materials Engineering, University College Dublin, Belfield, Dublin Ireland; 2 SeatTech Posture and Mobility Services, Enable Ireland, Dublin, Ireland

**Keywords:** Wheelchair, Cushion, Grasshopper, Scan to solid, Mesh editing

## Abstract

**Background:**

Custom orthoses are becoming more commonly prescribed for upper and lower limbs. They require some form of shape-capture of the body parts they will be in contact with, which generates an STL file that designers prepare for manufacturing. For larger devices such as custom-contoured wheelchair cushions, the STL created during shape-capture can contain hundreds of thousands of tessellations, making them difficult to alter and prepare for manufacturing using mesh-editing software. This study covers the development and testing of a mesh-to-surface workflow in a parametric computer-aided design software using its visual programming language such that STL files of custom wheelchair cushions can be efficiently converted into a parametric single surface.

**Methods:**

A volunteer in the clinical space with expertise in computer-aided design aided was interviewed to understand and document the current workflow for creating a single surface from an STL file of a custom wheelchair cushion. To understand the user needs of typical clinical workers with little computer-aided design experience, potential end-users of the process were tasked with completing the workflow and providing feedback during the experience. This feedback was used to automate part of the computer-aided design process using a visual programming tool, creating a new semi-automated workflow for mesh-to-surface translation. Both the original and semi-automated process were then evaluated by nine volunteers with varying levels of computer-aided design experience.

**Results:**

The semi-automated process showed a 37% reduction in the total number of steps required to convert an STL model to a parametric surface. Regardless of previous computer-aided design experience, volunteers completed the semi-automated workflow 31% faster on average than the manual workflow.

**Conclusions:**

The creation of a semi-automated process for creating a single parametric surface of a custom wheelchair cushion from an STL mesh makes mesh-to-surface conversion more efficient and more user-friendly to all, regardless of computer-aided design experience levels. The steps followed in this study may guide others in the development of their own mesh-to-surface tools in the wheelchair sector, as well as those creating other large custom prosthetic devices.

**Supplementary Information:**

The online version contains supplementary material available at 10.1186/s41205-022-00165-5.

## Introduction

Wheelchairs enable autonomy in many individuals across the world but sitting in a wheelchair for long periods can lead to pain, discomfort, and injuries such as pressure ulcers and spinal deformities. To prevent or correct such issues, customized wheelchair seating is manufactured for individual needs. Custom contoured seating (CCS) refers to customized seating that is based on an impression taken of a wheelchair user’s body and matches exactly to that shape. Custom wheelchair seating systems require shape capture of a surface moulded to the shape of the wheelchair user to design an effective personalised postural aid and pressure relieving intervention [1]. Methods to capture shapes include contact digitisers and non-contact scanning, including high-end time-of-flight or triangulation laser scanners as well as more affordable structured-light scanners [2].

Patient specific 3D printing typically begins with CT and MR images that are then segmented and converted to printable files [3]. However, there are a large portfolio of devices customized to a patient for which the Standard Tessellation File (STL) file is created via alternative strategies. These scanning techniques include laser trigonometric triangulation-based scanners and structured light scanners which both generate the digital recreation of an object or surface as a point cloud or a mesh object. Mesh objects are made up of tessellations that, when imported into a computer-aided design (CAD) program, are not editable because they are recognised as either a single entity or as thousands of small, individual surfaces. Because of this, if a 3D scan needs to be edited before manufacturing – to make a solid cushion or back support with the scanned surface, for example – the scan must be altered in a mesh-specific software or ‘converted’ into a CAD model and edited in a CAD environment. Mesh-specific software, like Meshmixer (Autodesk) or MeshLab (ISTI-CNR), offer sculpting as well as refinement and smoothing tools to alter the shape of a mesh object iteratively. Fernandez-Vicente et al*.* [4] developed an inexpensive, efficient method for scanning, designing, and 3D printing a thumb orthosis using only Meshmixer as the digital model creation tool. The new design and fabrication method suggested digitisation and 3D printing can cut costs and production time for orthosis producers. However, this process was not tested with clinical specialists, who may be less familiar with digital design tools and require more time and training to create an appropriate orthosis. Furthermore, mesh editing programs like Meshmixer do not offer parametric editing tools which would be used to alter measurable dimensions of a wheelchair cushion, such as the height or depth of the cushion for a better fit in a wheelchair base. Thus, for designing medical devices like wheelchair cushions that require more than the scanned shape in the model creation process [1], they do not meet user needs for digital alterations prior to manufacturing.

There are 3D printing software packages cleared by the United States Food and Drug Administration (FDA) for specific intended uses as part of a system that must include a specific 3D printer, material, and anatomy [5]. While the software is part of a cleared medical device, to date all such clearances have been for anatomic models and anatomic guides for parts 3D printed from CT, MR, and ultrasound images [6]. Such a software tool is not necessarily usable for other products due to this system-based method, but it suggests the need for further development 3D printing software packages and a broadening of their potential approved use cases to match the wide range of products that can be made using 3D printing.

To widen the range of potential products, we can look to CAD software packages which enable users to design and prepare models for manufacturing using direct editing or parametric modelling instead of mesh-only editing software. Some CAD software offer mesh editing tools similar to the mesh-specific software, and more and more CAD software producers are attempting to provide a means to convert mesh objects to non-uniform rational basis splines (NURBS) objects that are editable within a CAD environment. The NURBS object produced from such one-click conversions have as many surfaces as the mesh object had tessellations; for 3D scans, this can mean an object with tens of thousands of small surfaces that have to be manipulated when preparing the model for manufacturing, requiring large amounts of computer power [7]. A NURBS object with hundreds or thousands of surfaces is not a user-friendly object for those unfamiliar with CAD, such as the clinical teams prescribing and producing custom prosthetics, orthoses, and wheelchair seating.

This leads to a need for other ways to convert meshes to CAD models. Recent research has attempted to fill this void, specifically for custom prostheses and orthoses designed from body scans. Blaya et al*.* [8], Fernandez-Vicente et al*.* [4], Baronio et al*.* [9], Palousek et al*.* [10], and Paterson et al*.* [11] developed five different digital workflows for designing custom wrist orthoses or splints for3D printing. Works by Blaya et al*.* [8], Fernandez-Vicente et al*.* [4], and Paterson et al*.* [11] suggest that digital mesh models generated from 3D scans of wrists generate fewer tessellations than those of moulded cushions due to the size of wrist scans. This difference may make using mesh editing tools a less taxing task for wrist orthosis model generation than for CCS. However, these investigations did not evaluate the efficiency or user-friendliness of the digital workflows, nor did they evaluate the digital workflows with clinical experts to gauge the viability of the processes in a clinical setting.

Baronio et al*.* [9] developed a digital workflow that used both mesh editing software and NURBS conversion for CAD editing. This study deemed CAD editing necessary in their digital workflow due to the volumetric modelling needed to create an effective hand orthosis that was comfortable, light, and well-designed for its function. The authors discussed in their study how more user-friendly digital editing tools are needed in the clinical environment to use scanning and 3D printing to make custom orthoses, otherwise a CAD specialist is needed on the clinical team, implying that this may be a barrier to adoption for clinical teams. In their 2014 study on the design and 3D printing of custom hand-wrist orthoses in a clinical setting, Palousek et al*.* [10] similarly concluded that the digital workflow would require a CAD specialist or the automation of the workflow tasks that required the CAD specialist.

In 2018, Li and Tanaka [12] created a process that automated some of the CAD modelling tasks required in the digital design of custom wrist orthoses using Rhinoceros 3D and its visual programming plugin, Grasshopper (Rhinoceros 3D, McNeel & Associates). This study found the new workflow required 2 to 3 min of computer work compared to 20 min to 3 h of CAD modelling in the traditional digital splint process [13]. The authors show evidence in their study that the automated tasks created using Grasshopper could be used in the design of wearable orthotic devices and prostheses. In a separate study, Li and Tanaka [12] trialled their new process with five nursing students unfamiliar with CAD software. Their study indicated that an automated process with a simplified software interface enabled the nursing students to learn how to correctly use the process to design hand orthoses from 3D scans in less than one workday.

In this manuscript, a post-processing method to convert scans of custom back support and seat cushion surfaces into 3D CAD models was developed using Rhinoceros 3D’s Grasshopper plugin and trialled by potential clinical users of the process. Feedback from the trials was used to prototype a more user-friendly interface to the CAD environment and to simplify the conversion process further to make it more accessible to those with little to no CAD experience. The process used herein was based on Li and Tanaka’s work on the digital design of wrist orthoses in clinical settings [12]. The manuscript is divided into three main tasks: the documentation and assessment of the manual method for CCS design; the development of an automated workflow; and the evaluation of the proposed automated workflow.

## Methods

### Capture of existing manual workflow

**Fig. 1:**
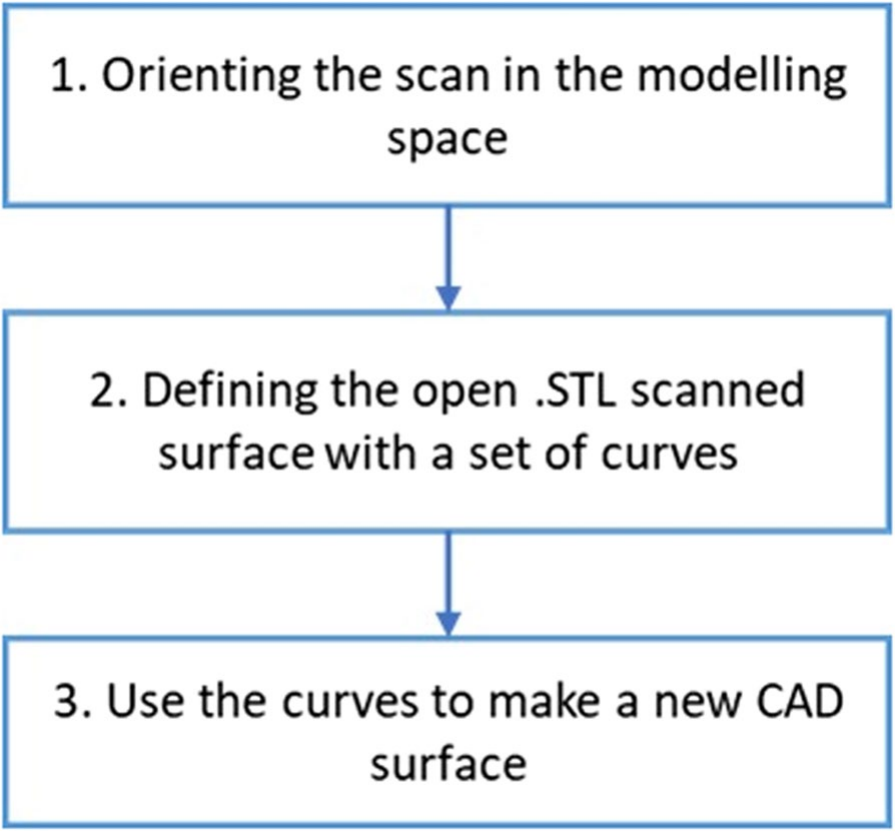
The three key actions in the workflow of converting an STL of a custom cushion scan into a CAD surface in Rhinoceros software

The first step in the development of an improved scan-to-print workflow was the capture and documentation of the existing process implemented by an expert clinical CAD user (volunteer C0). The researchers recorded the manual CAD method implemented by volunteer C0 in the prescription and manufacture of CCS systems. The original method employed Rhinoceros 3D (Version 6) and is documented in a step-by-step format [see Additional file 1]. Briefly, three key actions were identified in the process: orienting the scan in the modelling space; defining the STL surface with a set of curves; and using the curves to create a new CAD surface in the shape of the scan. These actions are shown in process order in Fig. 1.

#### User needs trial

Initial development work focussed on identifying user needs of the clinical team. For this reason, the original manual step-by-step process was provided to two potential clinical end-users to complete as a tutorial. The two clinical users (volunteers C1 and C2) had varying levels of CAD experience and worked in the wheelchair seating industry at the time that they completed the tutorials. Each volunteer gave live feedback during monitored trials of the manual process to capture user needs of the clinical team. The conversation during each test was open, to allow for any constructive feedback and each volunteer was asked a mixture of opened-ended questions [see Additional file 3].

Volunteer C1, a beginner CAD user, took approximately 21 min to complete the guide and had difficulty locating some tools and with using the ‘gumball’ tool. Specifically volunteer C1 could not locate the ‘line tool’ and the ‘surface from the network or curves’ tool. Other issues highlighted included (i) making the gridlines that would be projected onto the STL scan and (ii) selecting multiple lines in the modelling workspace. For (i), the volunteer did not know how many lines should be present; for (ii) the volunteer could not select multiple lines. Upon completing the trial, volunteer C1 described the process as too long and suggested that the process should be as automated as possible to simplify the process. They stated the process should take no longer than 15 min to complete.

Volunteer C2, who had no previous experience with any CAD tools, took approximately 38 min to complete the guide. Volunteer C2 struggled to understand the different viewpoints in the Rhinoceros software and had issues creating the gridlines. The volunteer described the process as “time-consuming” and stated that the ideal process should take less than 30 min. They suggested that a dialogue box on screen identifying the next steps would be helpful.

The key user needs identified from feedback during the trial are listed in Table 1. These (clinical) user needs were used to tailor the proposed automated process for the anticipated future users. Analysis of these user needs led to the determination of the following design requirements:

1. Automate as many steps as possible, including the orientation and creation of the surface step and the gridlines step.
2. Customise a toolbar or user interface to remove tools in Rhino’s interface that are not required and decrease overall time requirement.

**Table 1:** A tabulated list of user needs and their description for the custom cushion scan-to-CAD object process, discovered through the trial by potential end users of the original Rhino scan-to-CAD process

User Need	Description
The process is efficient	Time to complete process does not exceed 15 min
	There are no unnecessary steps in the process
The process is easy to use	The process is easy to learn for all users regardless of their CAD skill level
	The user interface is easy to understand
	The user interface does not cause confusion
	The user interface accommodates users with a range of skill levels
	The controls are easy to use and locate

### Semi-automated workflow development

This section details the development of a semi-automated workflow that utilizes Grasshopper in Rhinoceros 3D with the goal of satisfying the defined user needs gathered from the trial with clinical users. Grasshopper is a plug-in for Rhinoceros that enables the automation of many modelling actions through coded routines or the plug-in’s own visual programming language. This visual programming environment is not ideal for inexperienced CAD users to interact with when modelling, as it has its own set of skills to learn beyond the traditional CAD environment. However, Grasshopper has methods of creating separate Graphic User Interfaces (GUIs) with which users of the routine can interact instead of the visual programming environment of Grasshopper. To coincide with the user-need of “ease of use” in this project, the authors decided to use Grasshopper’s tools to create a simple user interface for the automated workflow.

**Fig. 2:**
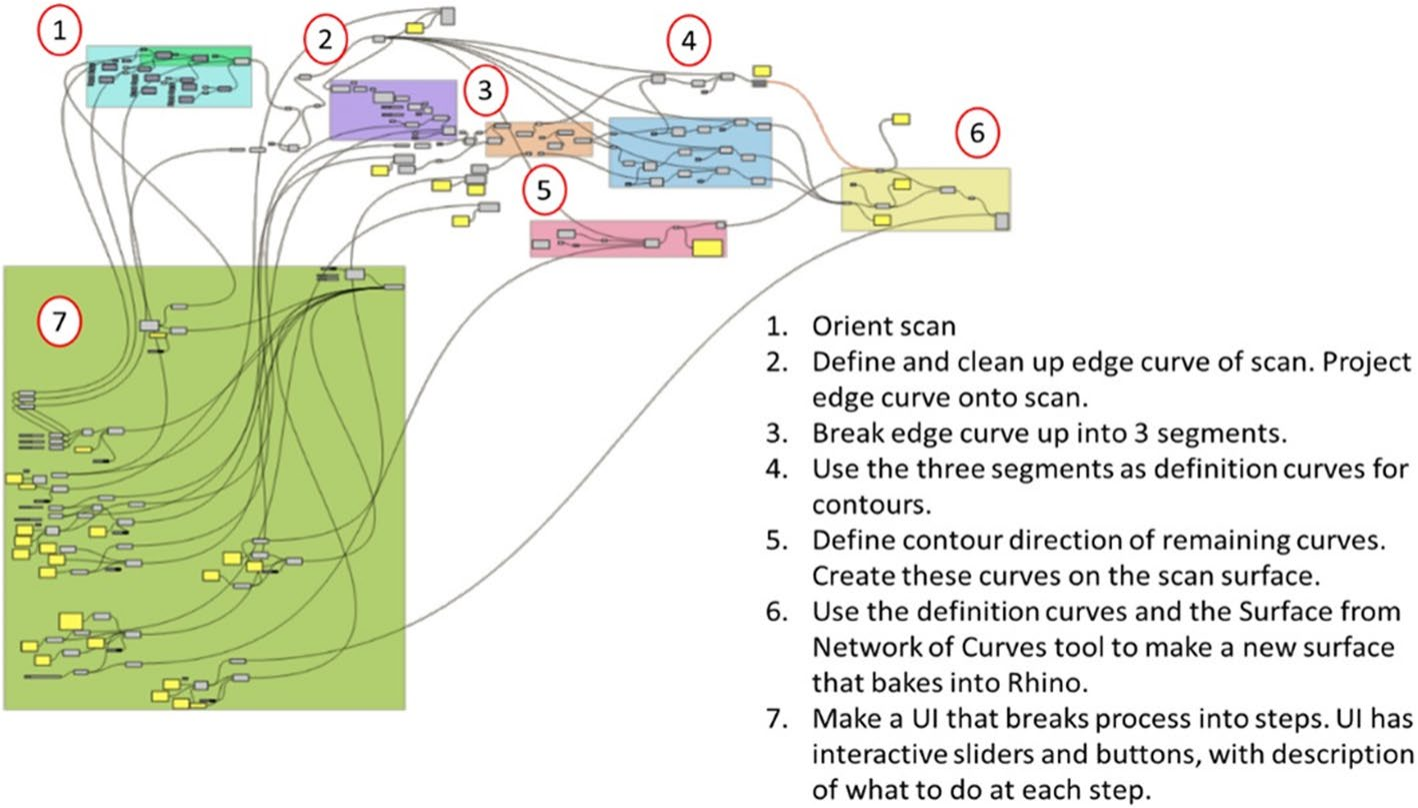
Picture of the Grasshopper program that automates the conversion of a STL cushion or back support scan into a CAD-editable surface. There are seven action blocks in the Grasshopper program numbered in the picture, with descriptions summarising what each block does

**Fig. 3:**
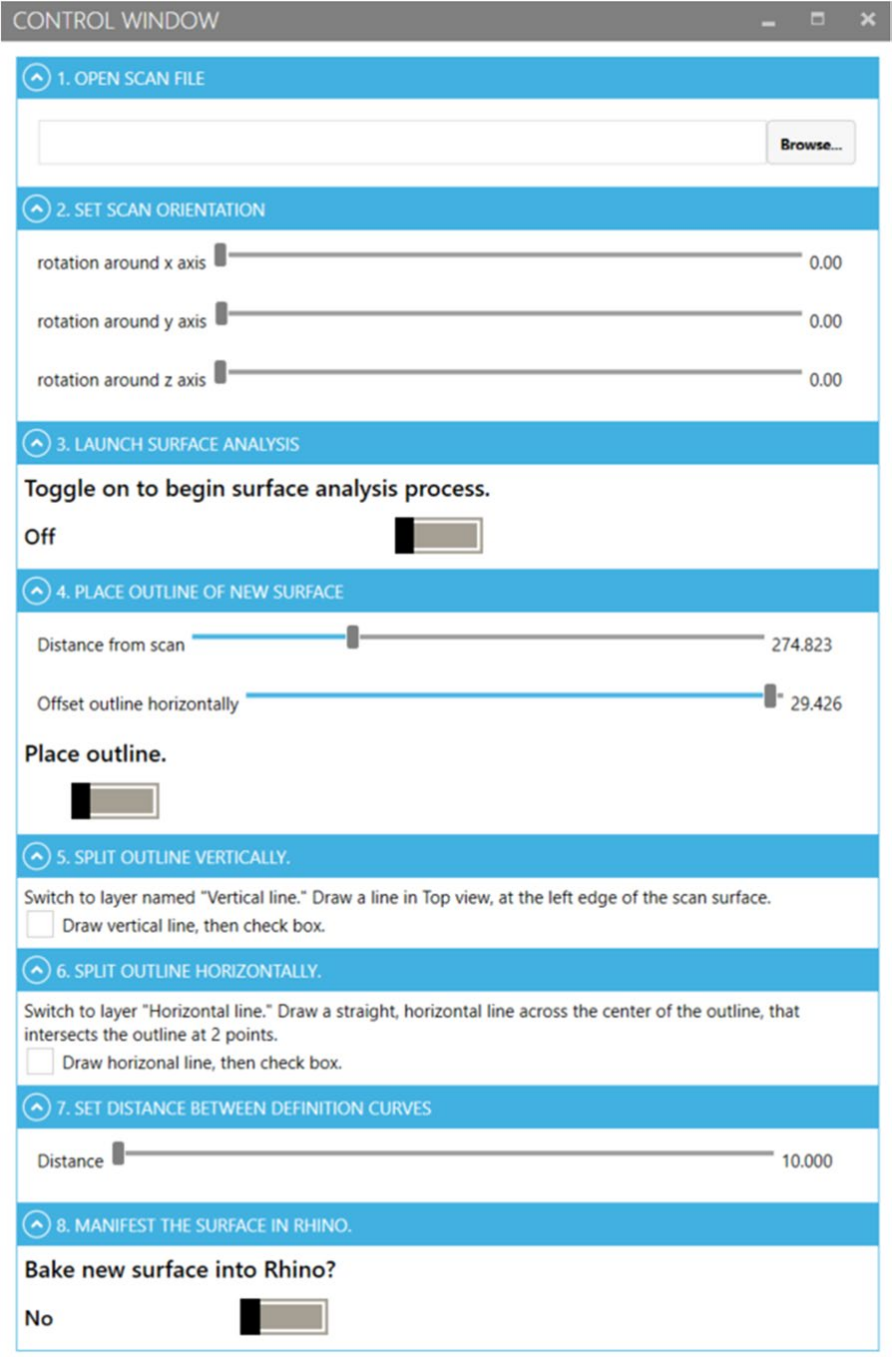
The user interface created using Grasshopper. This user interface allows users to set some modelling parameters to convert an STL cushion scan to a CAD-editable model through 8 steps

To create the user interface within Grasshopper, the modelling workflow first had to be translated into Grasshopper’s environment. The steps taken in the manual workflow were translated into Grasshopper’s visual programming language. Figure 2 shows the entire Grasshopper program, broken down into action blocks 1 through 7. Blocks 1 through 6 are directly related to making a CAD-editable model from the STL scan and block 7 creates the user interface that allows the user to set parameters of the model in blocks 1 through 6 without changing the Grasshopper file. The user interface is shown in Fig. 3.

The function of blocks 1 through 6 of the scan-to-CAD Grasshopper code, and how the user interface employs them, are further described in the following sections. A step-by-step guide for using the Grasshopper scan-to-CAD process with the custom user interface tool in Fig. 3 can be found in Additional file 2.

#### Block 1: Orienting the scan using Grasshopper

**Fig. 4:**
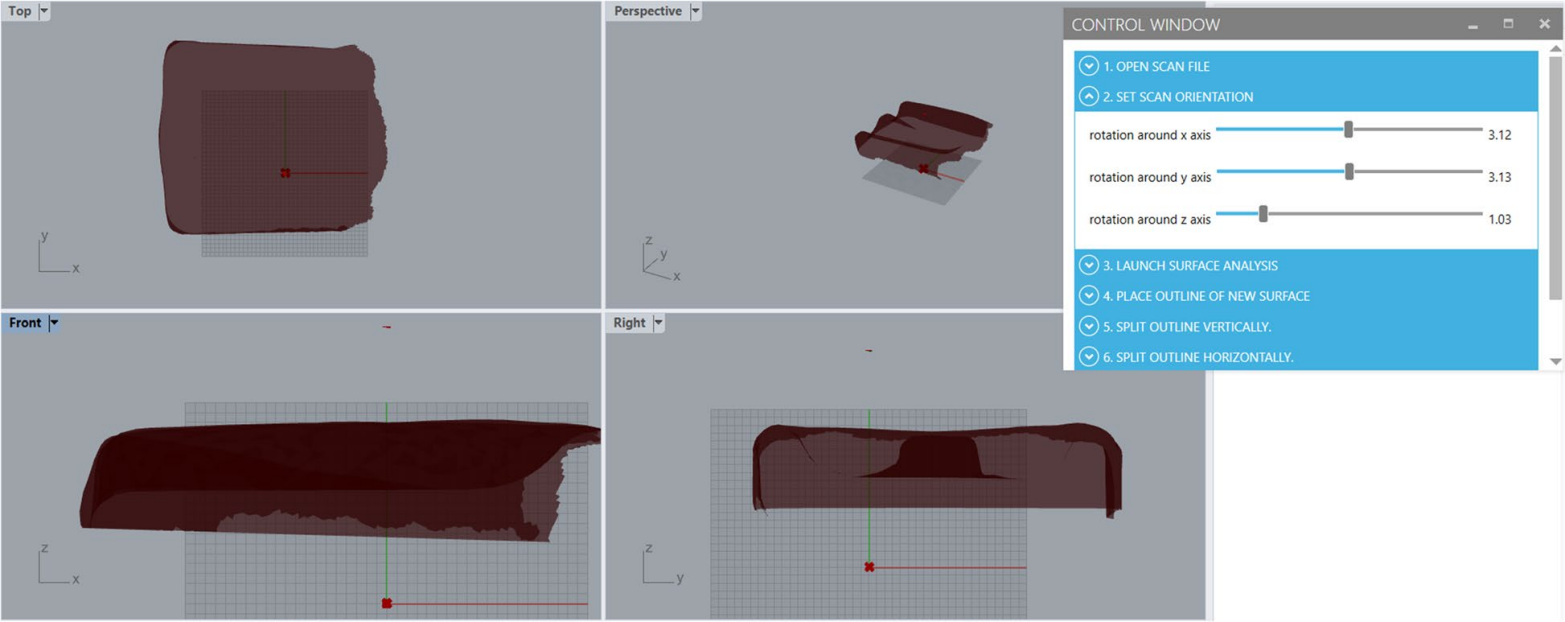
The correct orientation setting to begin the Grasshopper scan-to-CAD process. The Grasshopper program requires the user to orient the model such that the surface that conforms to the user can be seen in the XY-plane and that the front of the seat cushion or the bottom of the back support runs parallel to the Y-axis

The orientation of the STL scan in a 3D workspace dictates how it is translated into a 3D CAD model. The Grasshopper program requires the user to orient the model such that the surface that conforms to the user can be seen in the XY-plane and that the front of the seat cushion or the bottom of the back support runs parallel to the Y-axis, as shown in Fig. 4.

Block 1 enables the user to orient the STL cushion scan in the Rhinoceros modelling space regardless of the scan’s original orientation, one of the three key actions listed in Fig. 1. The programming block enables the user to rotate the scan about the X-, Y-, and Z-axes separately in such a way that any rotation is accounted for the other axes without the user needing to take action to ensure this. Each axis of rotation is defined in Grasshopper as the unit vector of its axis. The input of each rotation is connected to the user interface with sliding bars, enabling full rotation about each axis by the end user.

#### Block 2: Define and project edge curve

Once the correct orientation is set by the user, the scan needs to be “baked” into the Rhinoceros modelling workspace, so that it can interact with both Rhino and Grasshopper tools. Baking any model into Rhino also allows for saving and exporting of the model. The user must bake the oriented STL scan by toggling the switch in the “Launch surface analysis” tab to ON. As soon as this occurs, block 2 of the Grasshopper program begins its work.

**Fig. 5:**
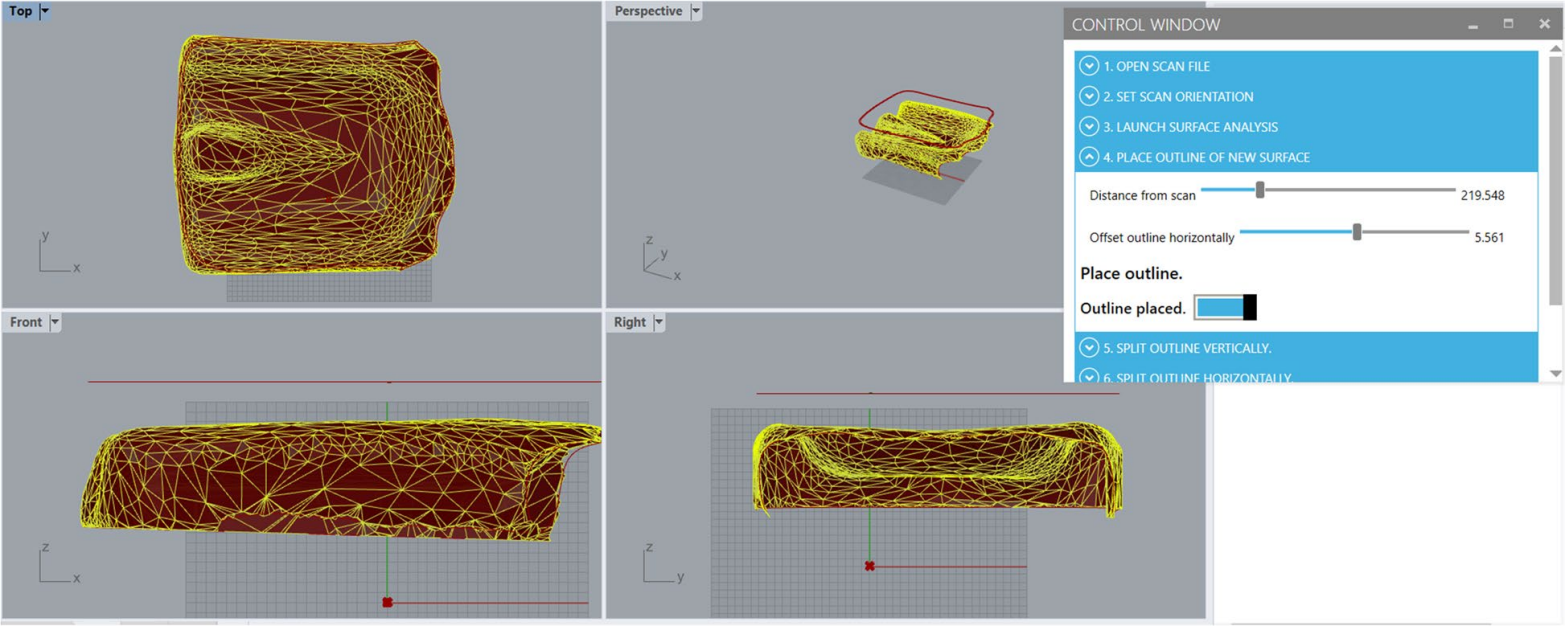
A depiction of how the polyline defining the outer edge of the cushion surface should be set using the Grasshopper scan-to-CAD program and its user interface

Block 2 of the Grasshopper program is set to capture the naked edges of the STL scan that has been baked into Rhino, turn the edges into a set of joined curves, and convert the curves into a polyline. This polyline is projected up to a plane above the model surface and copied. The copy of the polyline can be stretched or shrunk in the plane. The polyline copy is used to define a new outline of the CAD cushion surface, as shown in Fig. 5.

The user interface has 3 interactive tools in this step: two numerical sliders and a toggle switch. The first slider is used to move the plane up or down so that it is above the cushion surface, while the second slider stretches or shrinks the polyline copy that will define the new outer edges of the future CAD surface. The user must set the second polyline such that it resides inside the visible boundaries of the top of the cushion surface; if part of the polyline is wider than the cushion surface, it will not project onto the surface and the Grasshopper program will fail to move on to the next steps in the program. The toggle should be switched to ON once the position of the plane and polylines have been set as needed.

#### Block 3: Split edge curve

Once the outline has been defined and projected onto the STL model, this outline must be divided parallel to the X- and Y-axes to prepare the outline for final steps of the Grasshopper program. Block 3 divides the outline from Block 2 into three segments using lines created by the user in the Rhino workspace – one line in the X-direction and one line in the Y-direction. This is necessary to define the surface in the Rhino modelling workspace, instead of in tessellations used in the STL mesh format.

**Fig. 6:**
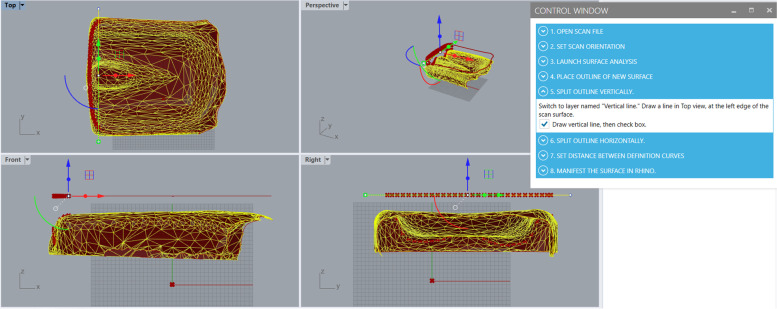
Using the Grasshopper scan-to-CAD program at step 5, where a vertical (X-direction) line is drawn by the user in the Rhino modelling workspace at the front end of a seat cushion or the bottom end of a back support. The red x’s seen projected from the hovering outline to the STL cushion show that the line intersects the outline as needed by the Grasshopper program

**Fig. 7:**
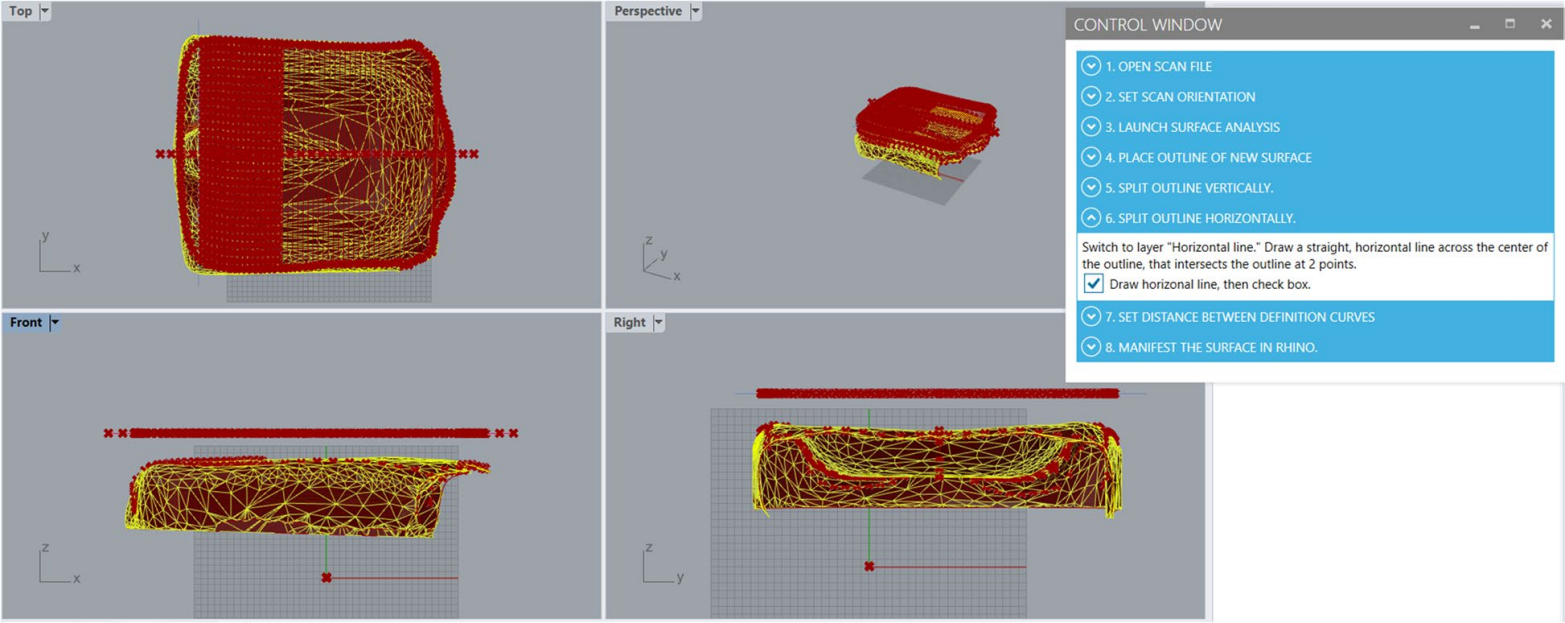
Using the Grasshopper scan-to-CAD program at step 6, where a horizonal (Y-direction) line is drawn by the user in the Rhino modelling workspace across the center of the STL cushion. The red x’s seen projected from the outline to the STL cushion show that the horizontal line intersects the outline as needed for the Grasshopper program

Block 3 is connected to the user interface tabs 5 and 6, shown respectively in use in Figs. 6 and 7. Tab 5 asks the user to split the outline once by drawing a vertical line in the Top viewport using Rhino’s *Line* tool. The vertical line must intersect the outline placed above the cushion; once this happens, red x’s appear along the outline showing nodes along the outline curve. Tab 6 of the user interface functions similarly to tab 5 but for a horizontal line in the Top viewport that splits the outline without red x’s in two. This horizontal line must intersect the outline, as shown in Fig. 7.

The newly split outline is now made up of 3 curves: one in the general X-direction and two in a perpendicular direction. These curves will be the first used in the rest of the Grasshopper program to build a contour map of the CAD surface from the STL cushion.

#### Block 4: Create contours in the Y-direction

The next step in the Grasshopper program consists of blocks 4 and 5 from Fig. 2, as they need to be concurrent to work correctly. Block 4 takes the three lines in the Y-direction –the horizontal line drawn by the user and the two lines created from the split by the user-drawn line—and rebuilds them so that they have an equal number of segments and control points, or nodes. The control points are then projected down onto the STL scan from the three horizontal lines, and these projected points are used to create NURBS curves along the shape of the scan, running in the Y-direction. This portion of the Grasshopper process does not require input from the user to run.

#### Blocks 5 and 6: Create contours and a surface from contours

Block 5 creates NURBS curves running in the X-direction that conform to the contours of the STL scan using Rhino’s *Contour* tool. This block correlates to tab 7 in the user interface in which the user is asked to use Rhino’s *Point* and *Line* tools to define in which direction the modelling workspace will make contours from the STL scan.

Once the contour direction has been defined by the user, the slider in tab 7 of the user interface is used to define the number of contour lines made and the distance between them in centimetres. The number of contours and space between them are used in block 6 of the Grasshopper scan-to-CAD program. Block 6 uses a Rhino software tool *Surface from a network of curves* that creates a surface using a network of intersecting curves.

**Fig. 8:**
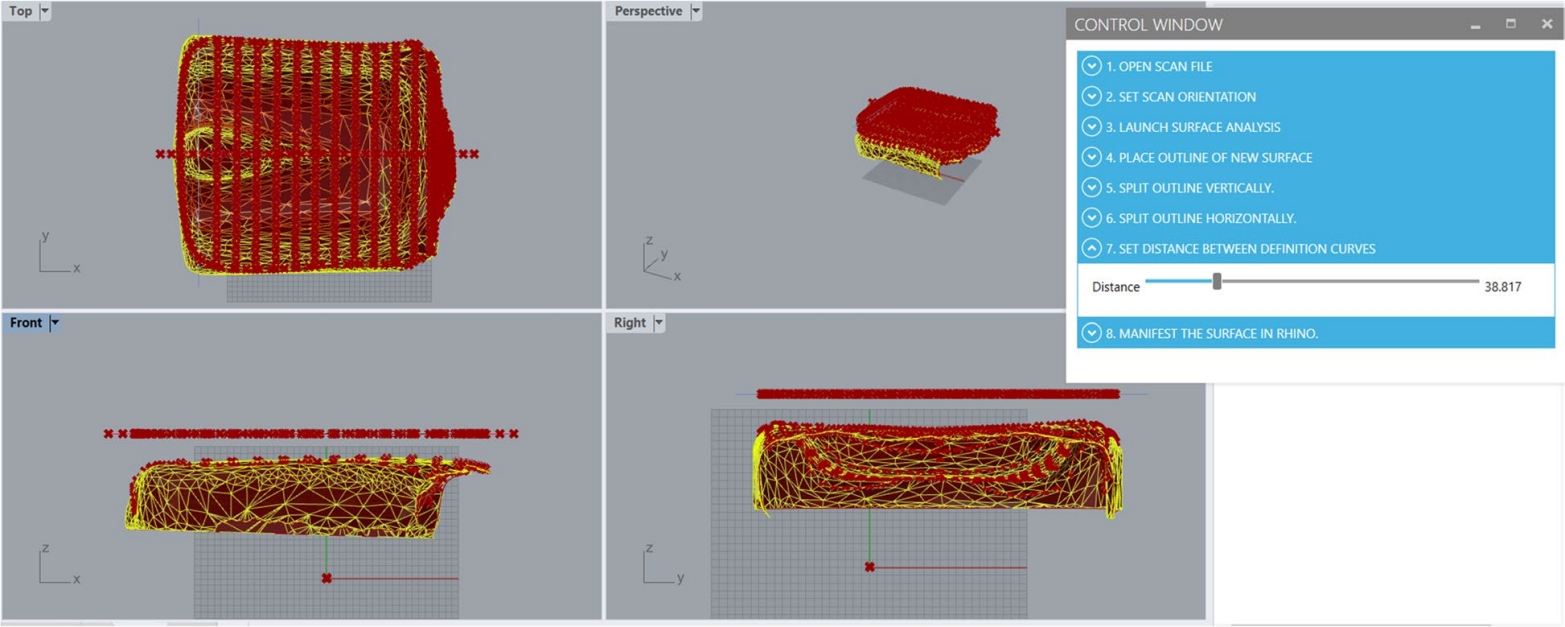
Tab 7 in the Grasshopper scan-to-CAD program user interface, showing the slider that adjusts the number of contour lines defining the cushion surface. A surface created from the contours is shown in the red areas overlapping the yellow mesh surface

The tool requires curves in the network to intersect each other not more than once, and to have all curves in one direction intersecting all curves in the other direction at least once. If these requirements are not met by the network of curves, a surface will not be created. To make the selection of curves useable in the Grasshopper program without requiring the user creating the surface to start the process over or conduct heavy computational editing, the Grasshopper scan-to-CAD program uses the slider tool in tab 7, shown in Fig. 8, to enable live definition of the network of curves used in the Rhino tool; the contours made in block 5 are the curves used in block 6, along with the curves made in blocks 3 and 4 of the program. The user of the custom program is able to see when a surface can be created from the network of curves when a red surface overlaps the imported STL mesh, visible in Fig. 8. There is not one correct set of curves that will create a surface; thus the slider enables choice for the user. Once a surface can be made, the user can bake the CAD surface into the Rhino modelling workspace by using a toggle in tab 8 of the user interface (Fig. 3).

#### Block 7: Creating a user interface for the process

The user interface was the last step in creating the automated process. Once the general steps that needed to be taken to create a CAD-editable surface from an STL scan were programmed into the Grasshopper environment, the *Human UI* Grasshopper plug-in was used to make an interactive user interface that leads users through the process. *Human UI* makes it so that parametric input tools in Grasshopper, such as sliders and Boolean toggle switches, can instead be placed in a separate window or tab from the Grasshopper environment and the parameters put in the user interface’s window are sent to the Grasshopper program live to update the model in the workspace.

### Workflow evaluation

Once both a manual CAD process and the semi-automated process – referred to as the Grasshopper process from here on – were created, a tutorial was written for the Grasshopper process [Additional file 2] similar to that written for the original CAD process [Additional file 1]. Nine volunteers were then recruited and tasked with trialling both processes by completing the tutorials for each process and recording the time it took for each volunteer to complete each process. Each volunteer self-reported their CAD experience level which were categorized using this study’s experience level organisation method and are listed in Table 2 below.

**Table 2:** Description of the different categories of CAD software experience. Representative numbers for each CAD level were assigned to each category for easy reference in the study

CAD Experience Level Category	Experience Level Description	Experience Level Representative Number	Volunteers at this Level
New user	No experience with any CAD software	0	V1, V2, V3
Beginner user	Learning one or more CAD software packages or last used CAD software more than 2 years before trial	1	V4, V5, V6
Intermediate user	Uses a CAD software between twice a year and once a month	2	V7, V8, V9

## Results

**Fig. 9:**
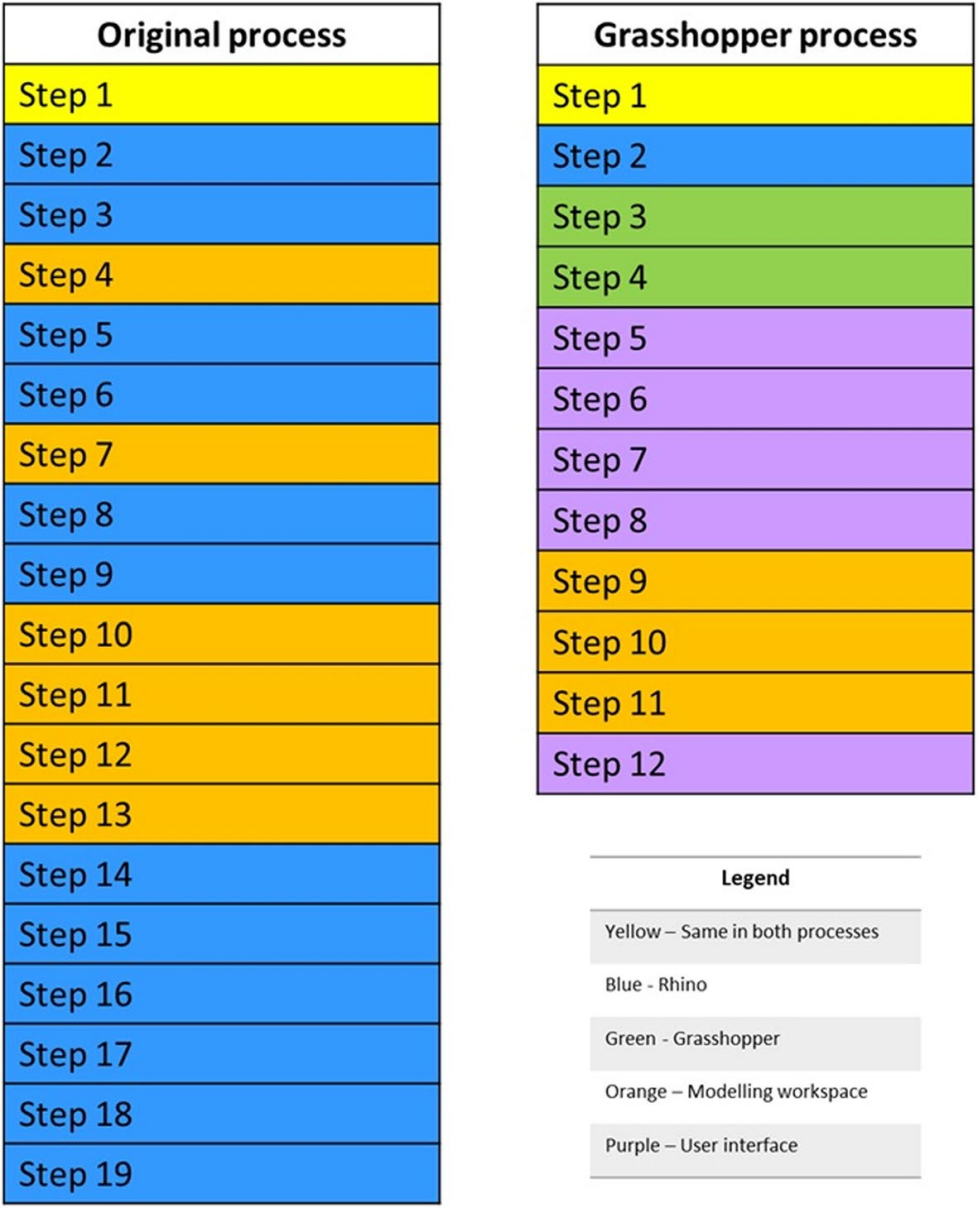
A comparison of the types of steps required in the original scan-to-CAD process in Rhino and the new process using Grasshopper. The type of each step is classified by what part of the software the user interacts with in the step

A total of 19 steps to convert an STL mesh model of a CCS cushion or back support into a CAD model were documented in the original process [see Additonal file 1]. The new Grasshopper scan-to-CAD program requires 12 steps to complete, a 37% reduction in the number of total steps to convert an STL scan to a CAD model. The types of steps required in each process are shown in Fig. 9, where the step type refers to what part of the software the user interacts with in the step. The figure shows that the number of steps in which the user must work in the modelling workspace, highlighted in orange in Fig. 9, is reduced by 50% in the Grasshopper process compared to the original process. This reduction suggests an easier process for users unfamiliar with CAD tools.

**Fig. 10:**
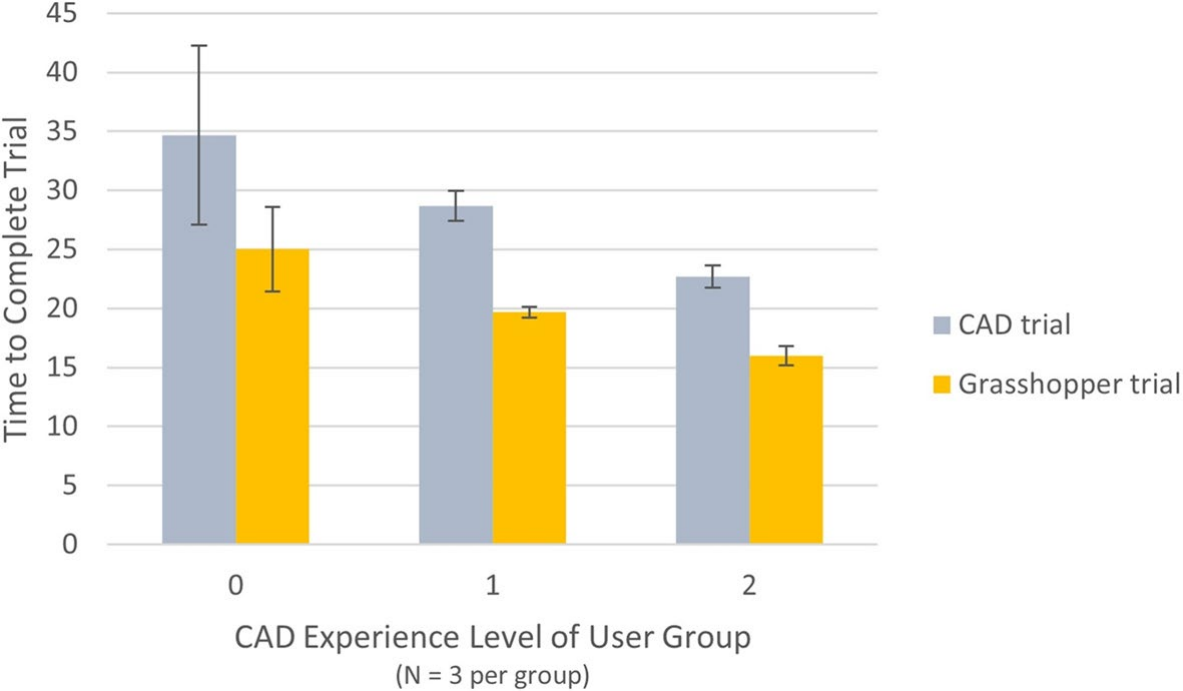
Time to complete each process trial, grouped and averaged (*N* = 3 per group) by CAD experience level of the volunteers, with standard deviation bars shown

Results comparing the time taken for each volunteer to complete the two modelling processes are shown in Fig. 10 below. The figure shows a decrease in the time to complete trials as the CAD experience level of the user increased, regardless of the modelling process. Although this result is expected, it confirms that the modelling process and tutorials are logical to engineering users. Results also indicated that the Grasshopper process decreased the time to convert an STL scan to a CAD surface from an overall average of 29 min to an average of 20 min for all volunteers (*N* = 9). This change equates to a 31% decrease in the time needed to complete the modelling process, regardless of CAD skill level. Additionally, within each CAD experience group there was a decrease in the time to complete the modelling process when following the Grasshopper method as compared to the original modelling process. These results suggest that using the Grasshopper method in the creation of custom wheelchair cushions would reduce the time needed to complete the digital portion of the manufacturing process.

## Discussion

Results from both clinical users and the volunteer comparison trials of this study suggest that a manual process is valid though not the optimal method, especially for inexperienced and beginner CAD users. Feedback from medium- to high-skill CAD users suggest that the Grasshopper process is more accessible and easier to use than the manual conversion process using only Rhino software and its modelling workspace. The scan-to-CAD process cannot be completely automated due to the variance in the shape of input scans, thus all users new to the process will find tutorials on how to use the Grasshopper process useful. Results from Li and Tanaka’s work on custom wrist splint design suggest that users of all skill levels can learn a custom scan preparation process with sufficient training [12]. Those implementing such custom methods into their practice will benefit from training their users, and training only the use of the custom process is shorter than that required to be an expert Rhino or other CAD software user. Training was not performed with the second cohort of volunteers in this study; the results from these volunteers still indicate that the tutorials for both processes are sufficient to complete the modelling process, though the Grasshopper method requires less time to complete regardless of CAD skill level.

The quantitative results support the positive feedback on the Grasshopper process from clinical users. The decrease in time to convert the STL to a CAD surface is similar to the percent decrease in the number of steps, suggesting that the custom user interface in the Grasshopper scan-to-CAD process is less confusing or less overwhelming than just interacting with the Rhino interface and its CAD tools. As with any skill, it is expected that the average time to complete the Grasshopper process would decrease further as users become more familiar with the method. The time decrease could enable faster production per seating system and increased production capacity for any clinical team producing custom seating, enabling further outreach to wheelchair users.

The user interface could be further optimised, for example by creating a plugin for Rhino using C/C +  + with its own unique and streamlined GUI, though that is outside the scope of this study. This study was designed to demonstrate whether it is feasible for clinical teams without CAD experts or skilled programmers to prepare STL scans for 3D printing or further CAD manipulation. The semi-automated process outlined here has been trialled with non-expert users of CAD software and has recorded an average time to independent completion of 20.2 min, thereby demonstrating feasibility. The process outlined here to create a custom user interface and scan-to-CAD conversion process can be used by other researchers, manufacturers, and clinical teams as a baseline process to prepare custom orthoses and seating for different manufacturing processes. Detailing the Grasshopper process here allows for customisation and alteration of the method as needed for other applications. As the Rhino software and Grasshopper plug-in are updated and new tools are created, the Grasshopper scan-to-CAD process can be changed to suit the desires and needs of users.

## Conclusion

The goal of the modelling process in this study was to convert an STL format scan of a custom moulded seat or back support into a CAD object, editable not only in Rhino but in other CAD software packages if desired. The manual workflow accomplishes this task but had room for improvement for use in a clinical setting with users who may not be familiar with CAD software tools. As more clinical teams turn to different forms of scanning to make custom wheelchair seating, having tools to fit the needs of the team and the wheelchair user is key to increasing use of high-end technology in clinical settings and orthosis manufacturing. Publishing details of processes such as the semi-automated process described herein further enables the use of new technology by non-profits and low-budget teams.

It also allows other teams the ability to customise a digital workflow for processing scans for other 3D printed medical product design applications. Any process that requires an STL model to be parametrically edited after a scan, such as upper and lower limb prosthetic design or moulds for custom dental devices, could take the building blocks of the Grasshopper tool created and tested in this paper and restructure them to suit the needs of the product. Devices in the medical industry are already benefiting from moving to 3D printing as a manufacturing method [14, 15]. To make custom 3D printed products more widely available to patients, creating and supplying software tools that ease the pain of device design without majorly increasing costs or design difficulty is key.

## Supplementary Information

**Additional file 1.**

**Additional file 2.**

**Additional file 3.**

## Data Availability

The datasets generated from the study are available upon reasonable request in an anonymized, quantitative form to protect the privacy of participants. Step-by-step guides used by participants in the study are supplemented as Appendices to the article. The Rhinoceros 3D plug-in tool developed through the work in this study is available upon reasonable request.

## Abbreviations

CCS: Custom-contoured seating
STL: Standard tessellation language
CAD: Computer-aided design
NURBS: Non-uniform rational basis splines
GUI: Graphic user interface
