# Supplementary material for: Development and evaluation of a facile mesh-to-surface tool for customised wheelchair cushions
Source: 3D Print Med. 2023 Feb 13;9:3. doi: 10.1186/s41205-022-00165-5 (PMC9926538; doi:10.1186/s41205-022-00165-5)
Supplement: Supplementary file 1 — Additional file 1. [file 41205_2022_165_MOESM1_ESM.docx]

Additional file 1

Steps to make a custom cushion scan CNC-able:

1. Open Rhino3D software
2. Go to File → Import → pick the scan file you want to edit. It should now be loaded and visible in the Rhino software.


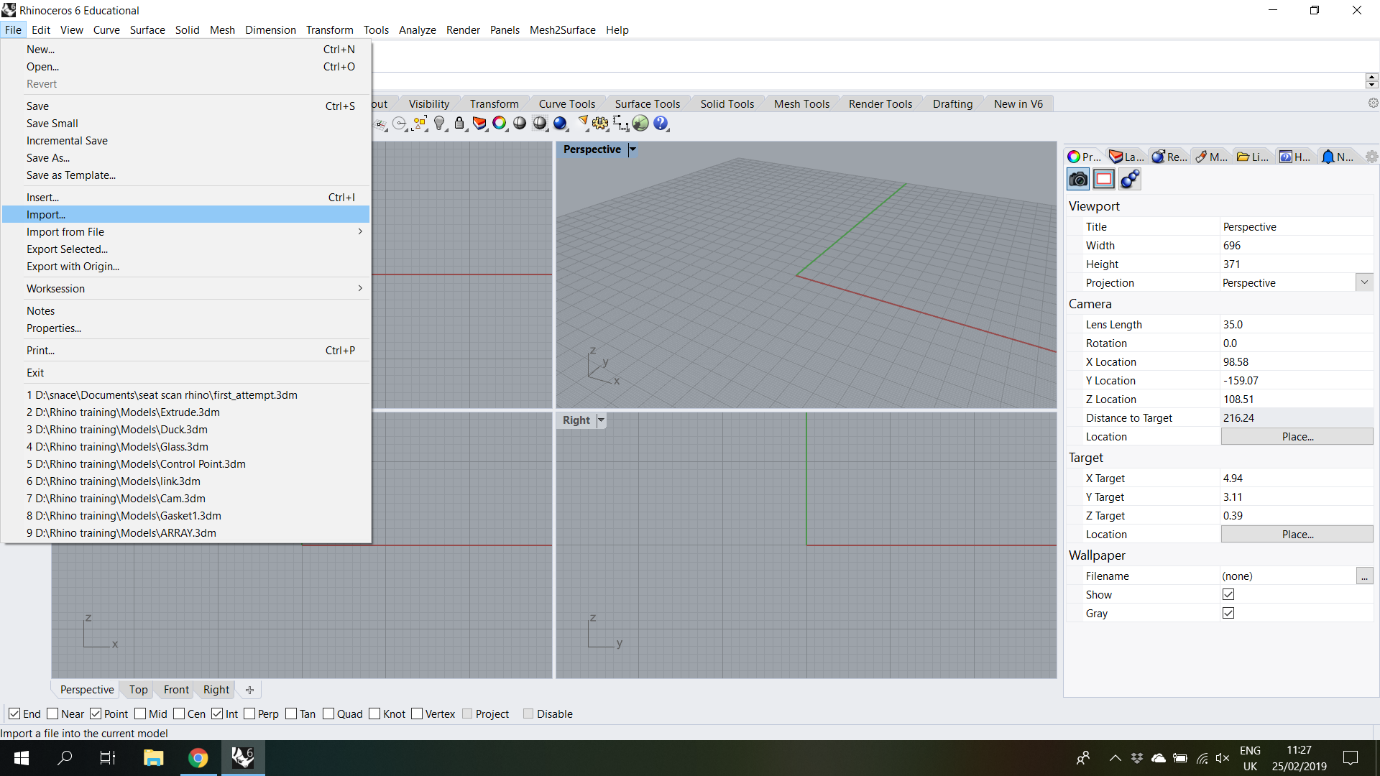


1. Double-click *Perspective* viewport title box. This expands the screen to only the *Perspective* viewport.
2.
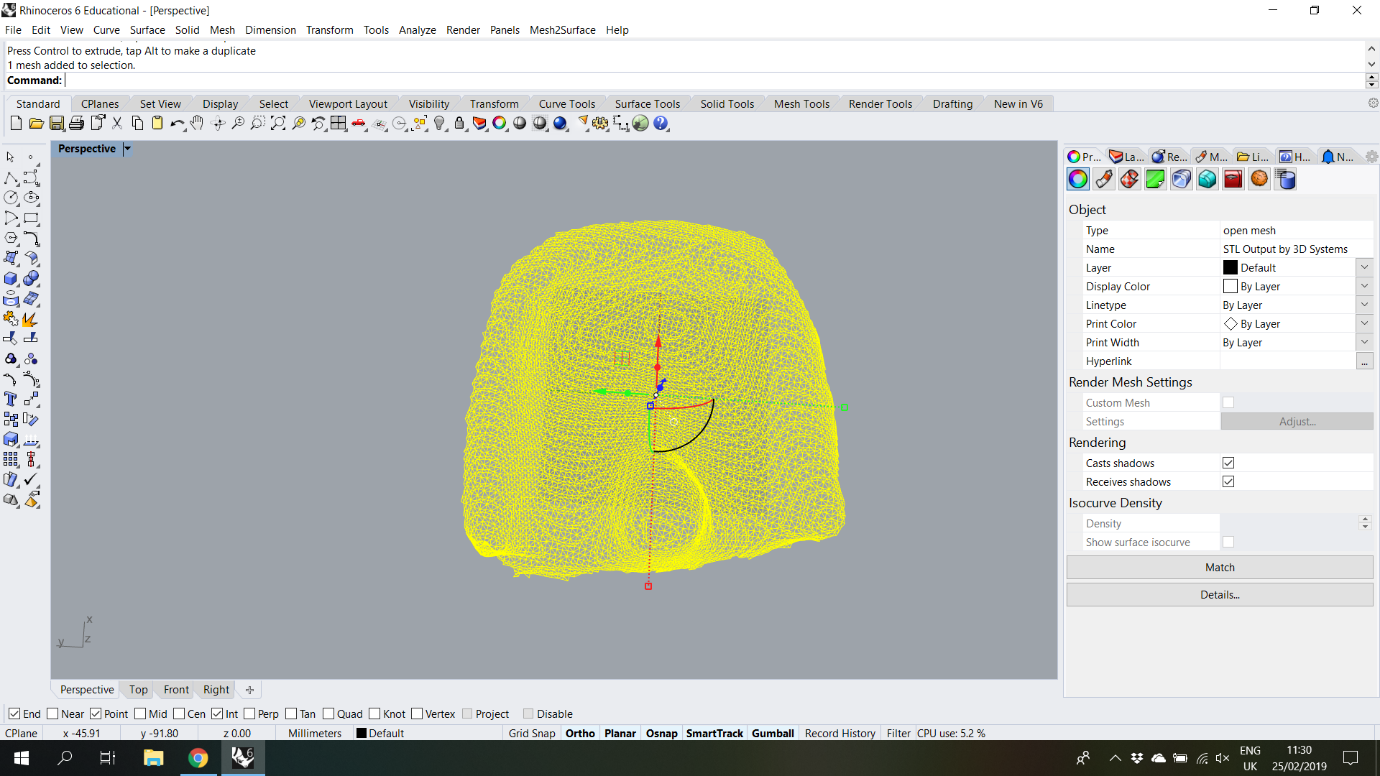
Orient the scan (rotate, move) using the Gumball cursor as needed so that it is aligned properly in the XYZ planes. This will make it easier to work with later, and ensures the CNC will be carved in the right orientation later

| **FYI: Gumball Controls are as follows:**   - **Left-mouse click + hold an arrow + drag → moves object in that arrow direction** - **Left-mouse click + hold an arc + drag → rotates object in that plane** - **Left-mouse click + hold a square + drag → scales the object in that direction** - **Left-mouse click + hold a square + Shift + drag → scales entire object in 3D** - **Press + hold Ctrl during any move, scale, or rotation → extrudes a planar curve or surface of the object** |
| --- |


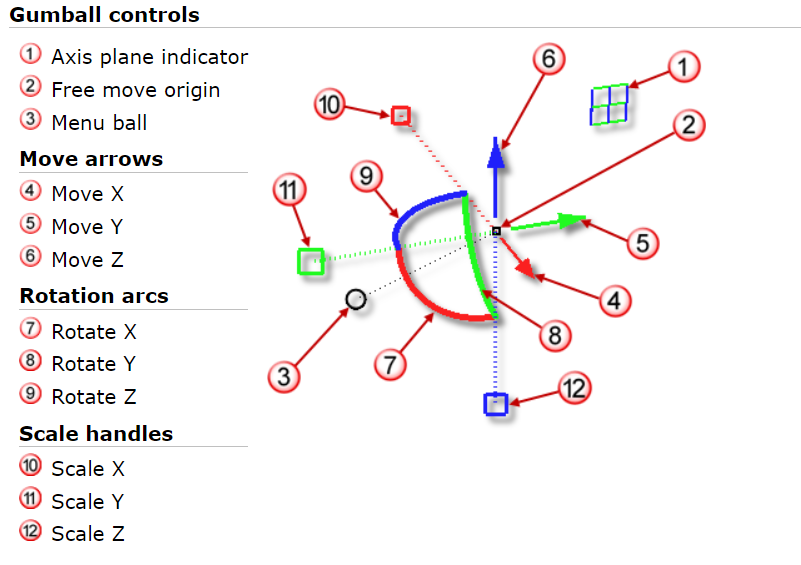


1. In the right tab, open the *Layers* port. Rename a layer “Original scan” by double-clicking a layer name and typing into it.


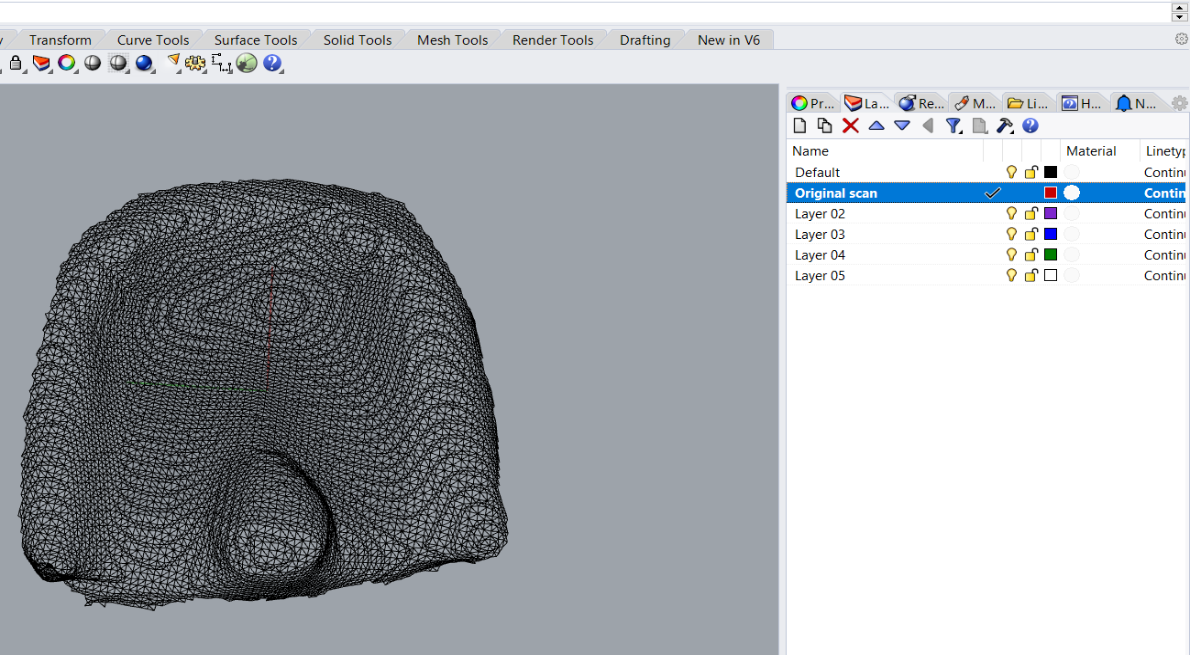


- 1. Select the scan object (left-click the scan object so that it is highlighted).
  2. Right-click the layer name *Original scan* → “Change object layer”
     1. This moves the scan to this layer, which will change the appearance colour of the object in Rhino. You can now easily hide and show the scan by clicking the lightbulb for its layer tab.

| **FYI: The active layer displays in bold and has a check-mark next to its name. Double-clicking a layer will make it the active layer to work on. Working with layers makes it easier to see and work with multiple objects on the screen.** |
| --- |

1. Rename a different layer *Grid lines*. Make this the active layer.
2. Draw a set of grid lines across the surface of the scan.
   1. Switch to the *Top* viewport. You can easily do this by clicking Top at the bottom-left corner of the viewport, where all of the views are listed. You may need to zoom out (using the mouse scroll wheel) to see the entire scan in the new view.


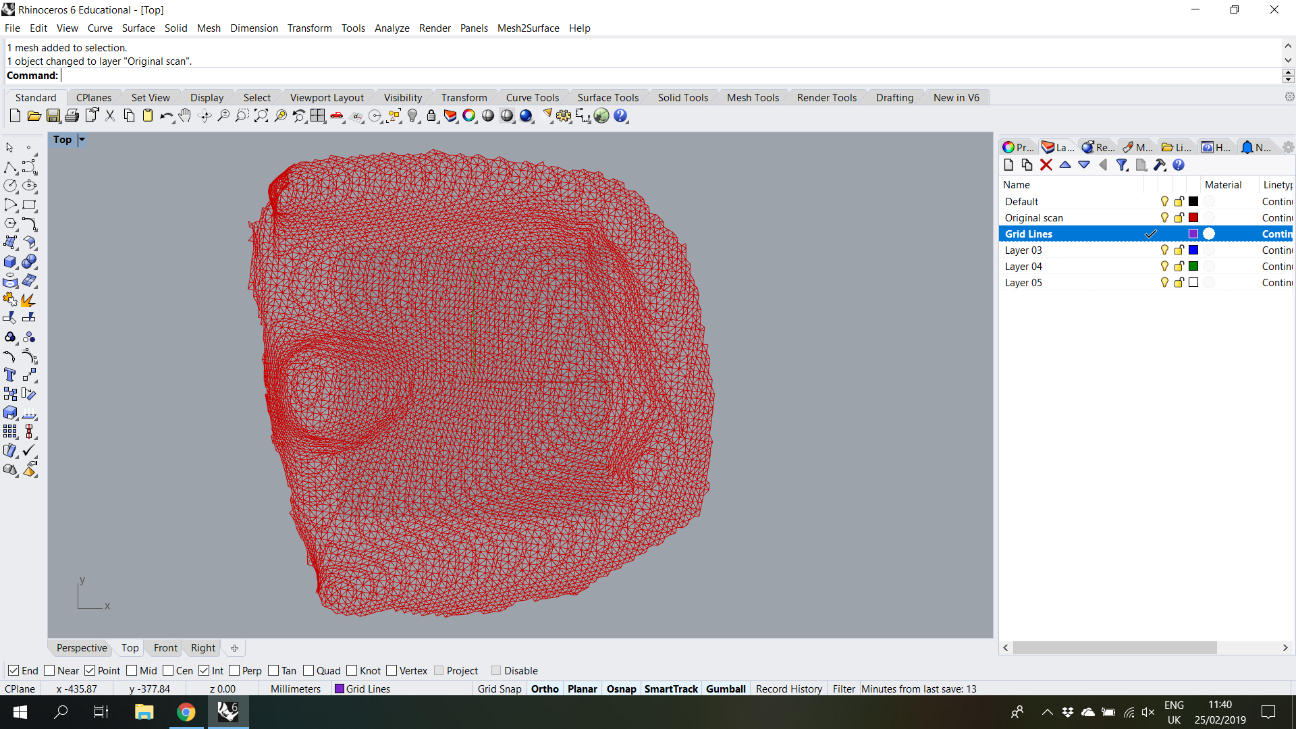


- 1.
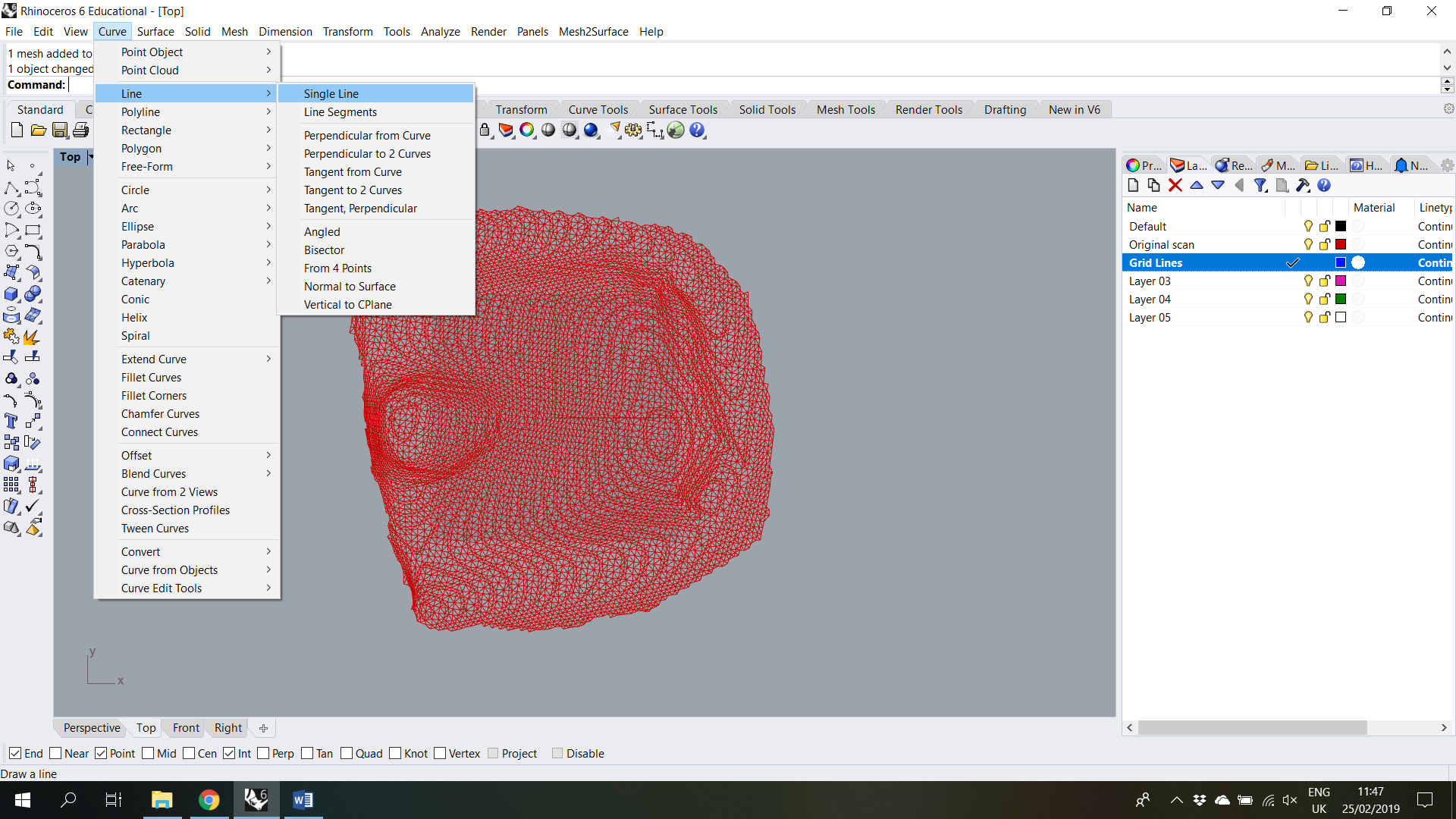
Select the Curve tab → Line → Single line. Left-click two points on the screen to make a horizontal or vertical line that spans across the scan.
  2.
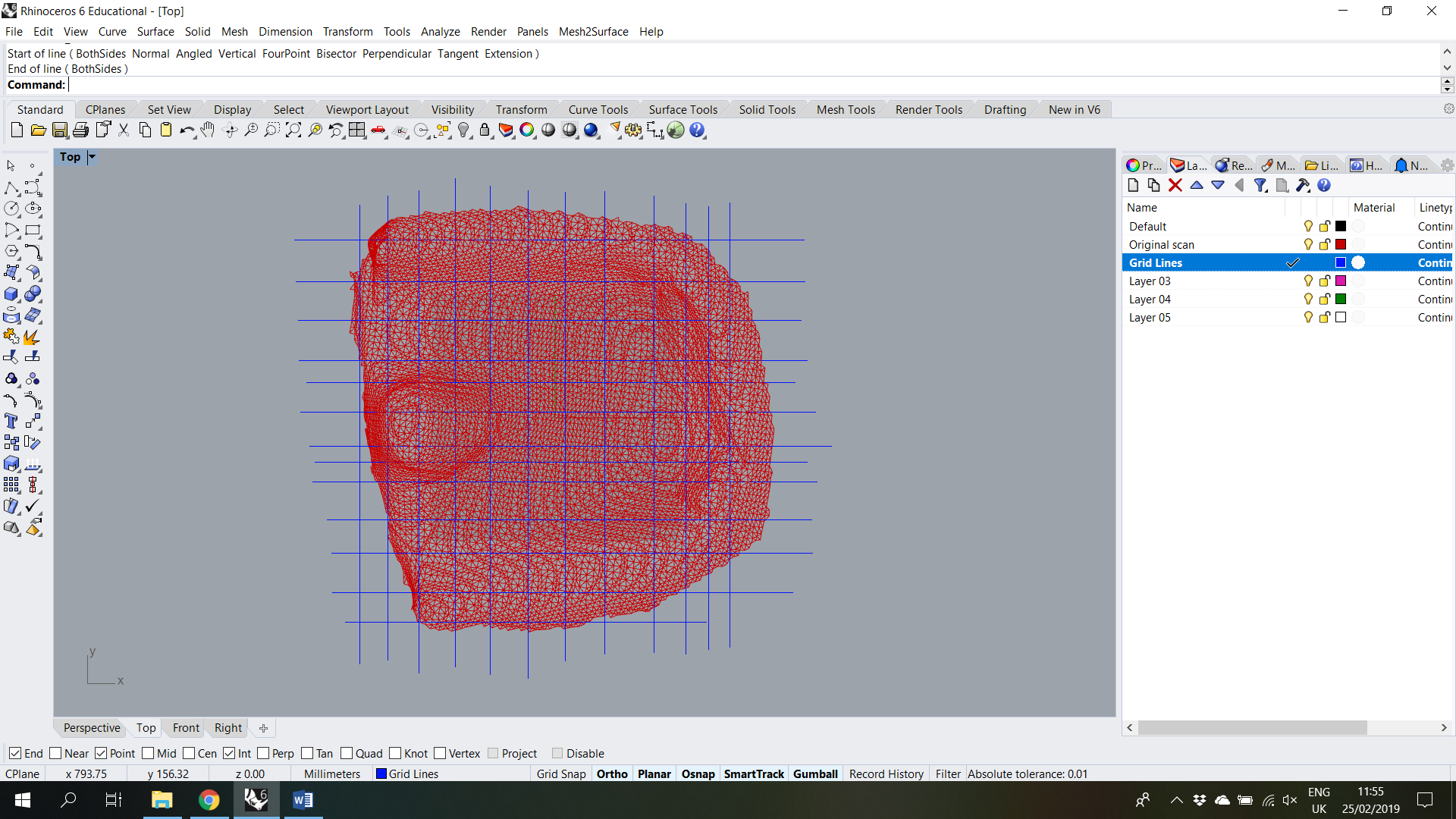
Repeat the Single line command, making first the horizontal line set and then the vertical line set (or vice versa), until a grid is made.
  3. Check that the grid lines do not intersect the scan by switching to the *Right* or *Front* view. If they do:
     1. Edit → Select objects → Lines. All the grid lines will be highlighted yellow when selected.
     2.
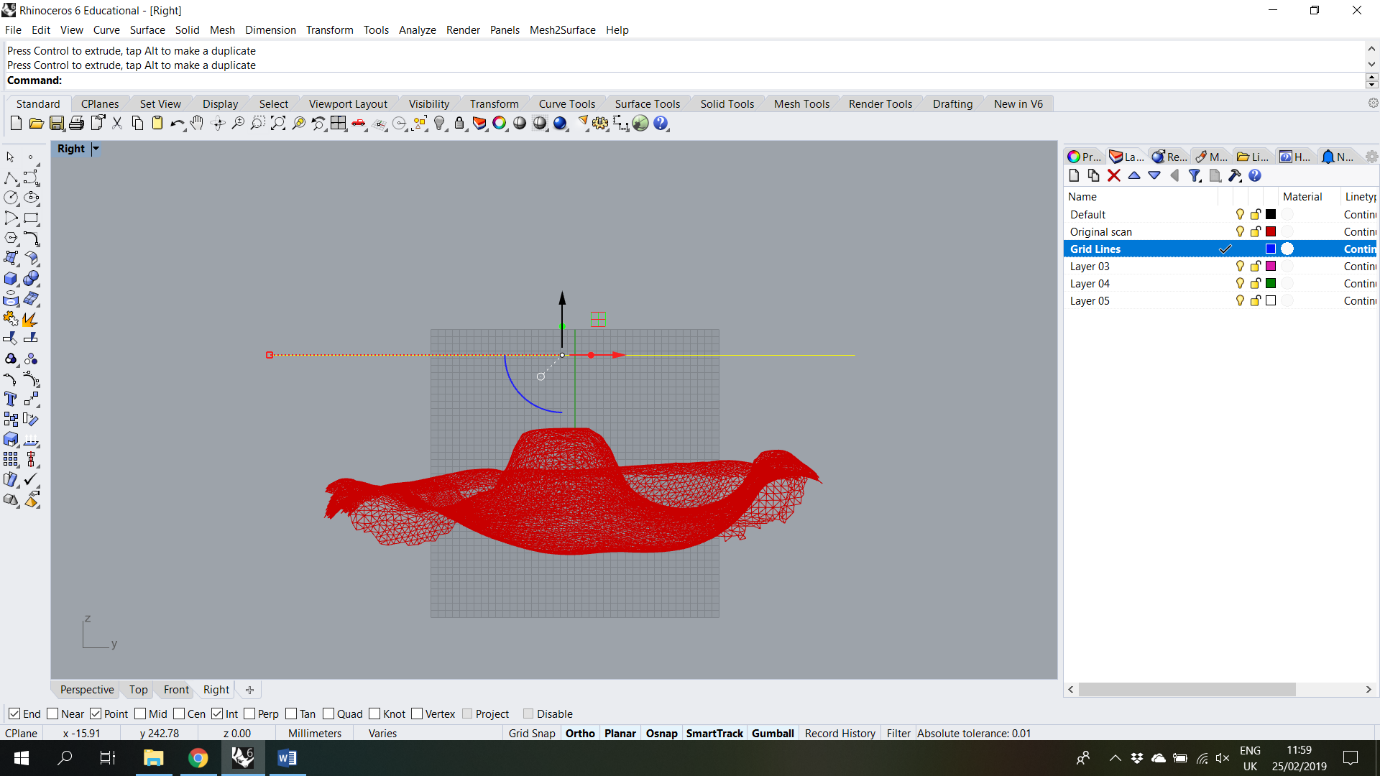

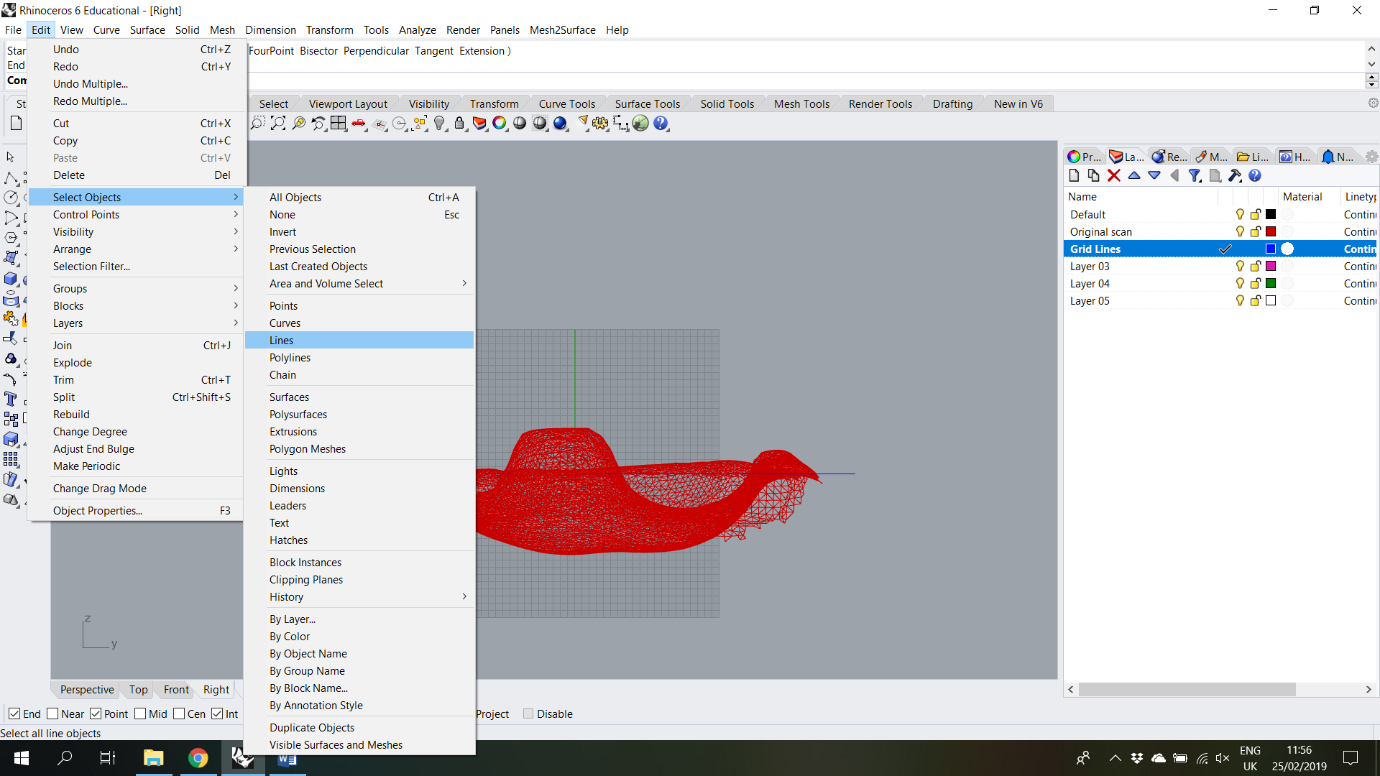
Using the Gumball cursors, move the set of grid lines straight above the contoured surface (click an arrow and drag).

1. Switch back to *Perspective* or your preferred viewport.
2. Rename a different layer *Projected curves*. Make this the active layer.
3. Select all of the horizontal grid lines in one direction. (Hold Shift and Left-mouse click to select multiple objects.)
4. Type “Project” into the command line. Press Enter.
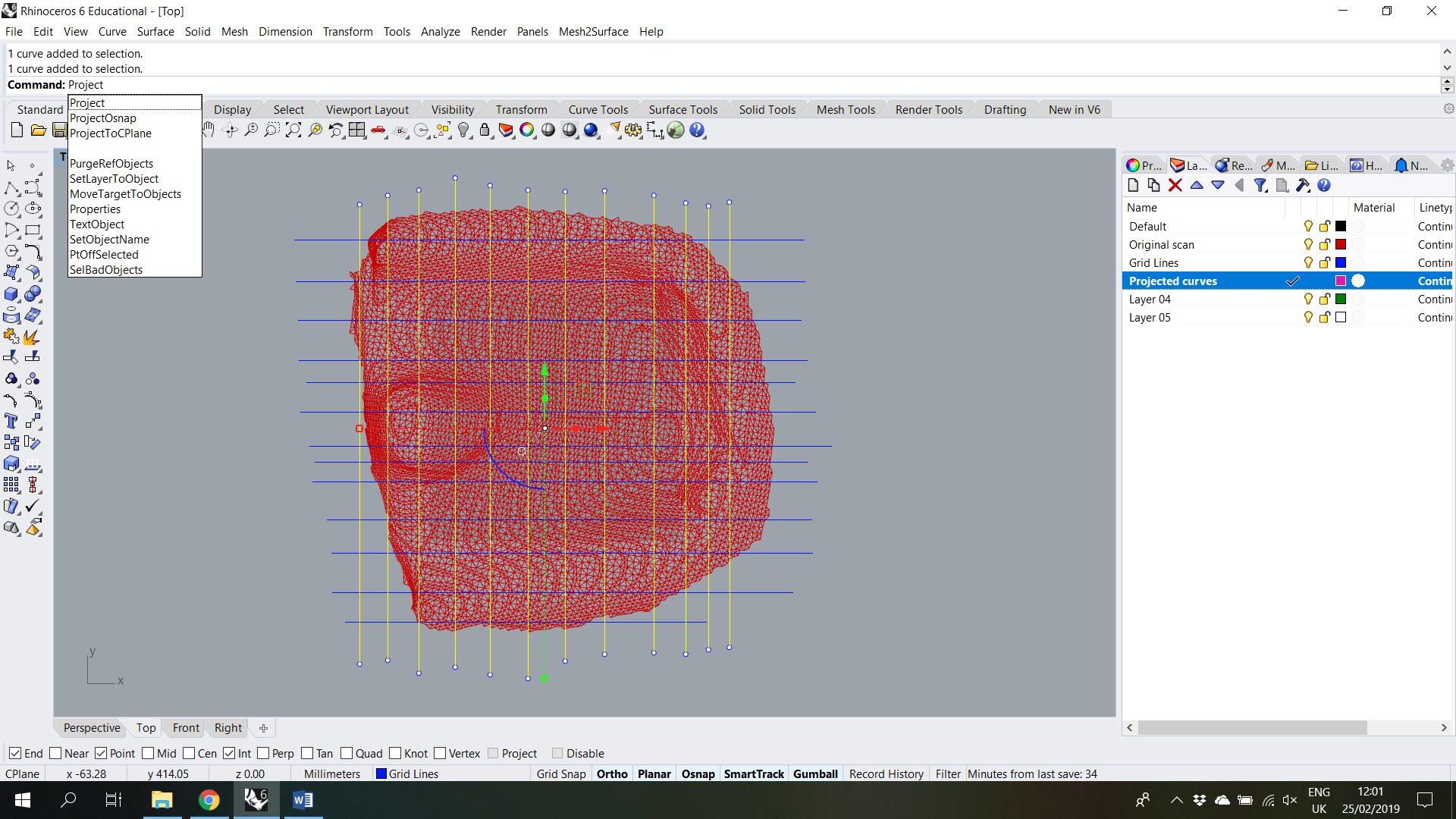

5.
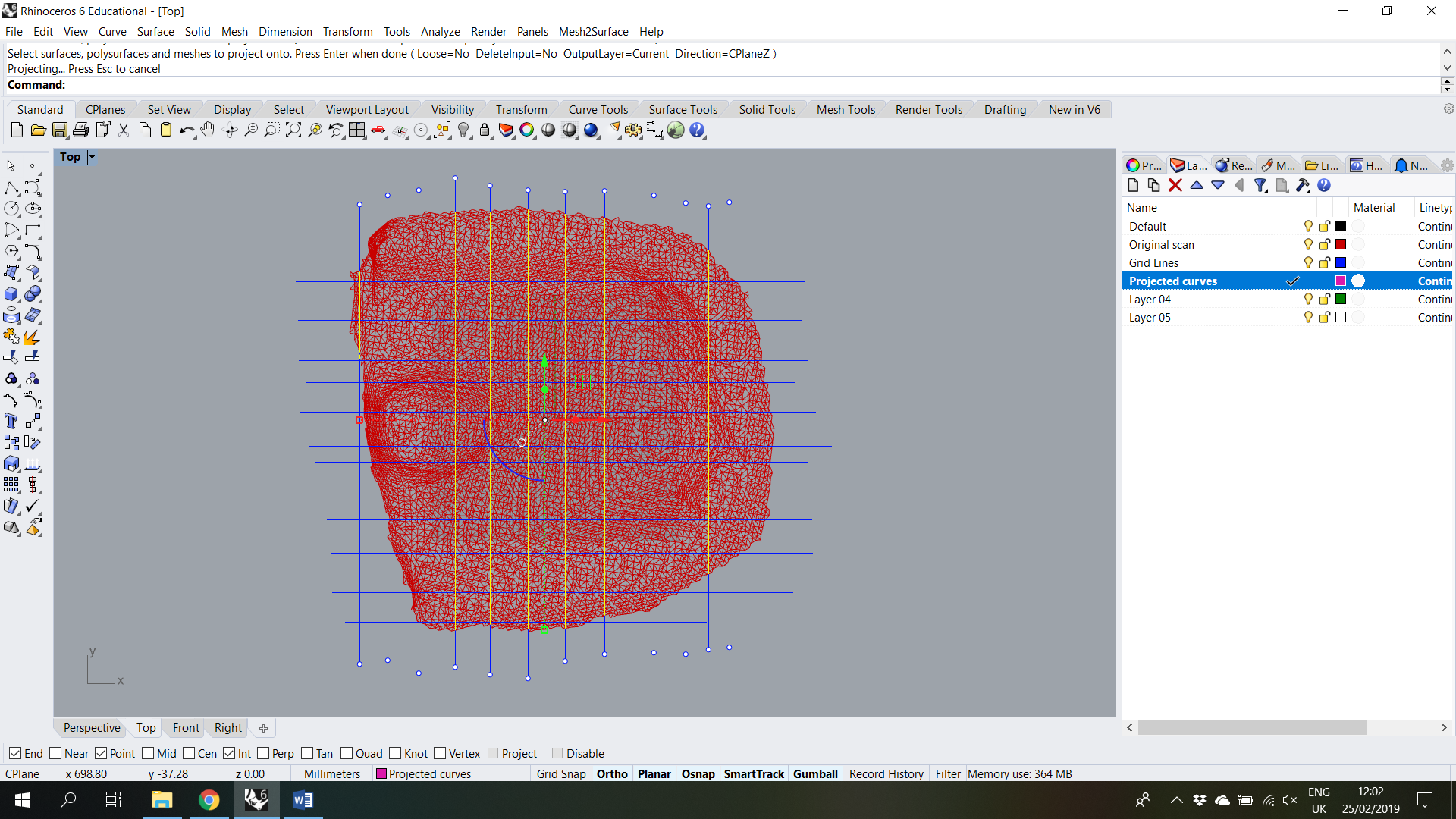
Select the scan as the surface or object to project onto. Press enter. A new set of curves now follow the surface of the scan.
6. Repeat Steps 10-12 for the vertical grid lines.
7. Window select all of the grid lines (not the curves on the cushion). This may be easiest to do from the *Front* or *Right* viewport. Then go to Edit → Visibility → Hide selected. **Or** turn the lightbulb for the “Grid lines” layer off. This will hide the grid lines from view so that they cannot be selected in the following steps. They can be turned back on at any time showing the lines or turning the lightbulb for the layer on.
8. Edit → Select objects → Curves. This will highlight all of the projected curves on the scan.


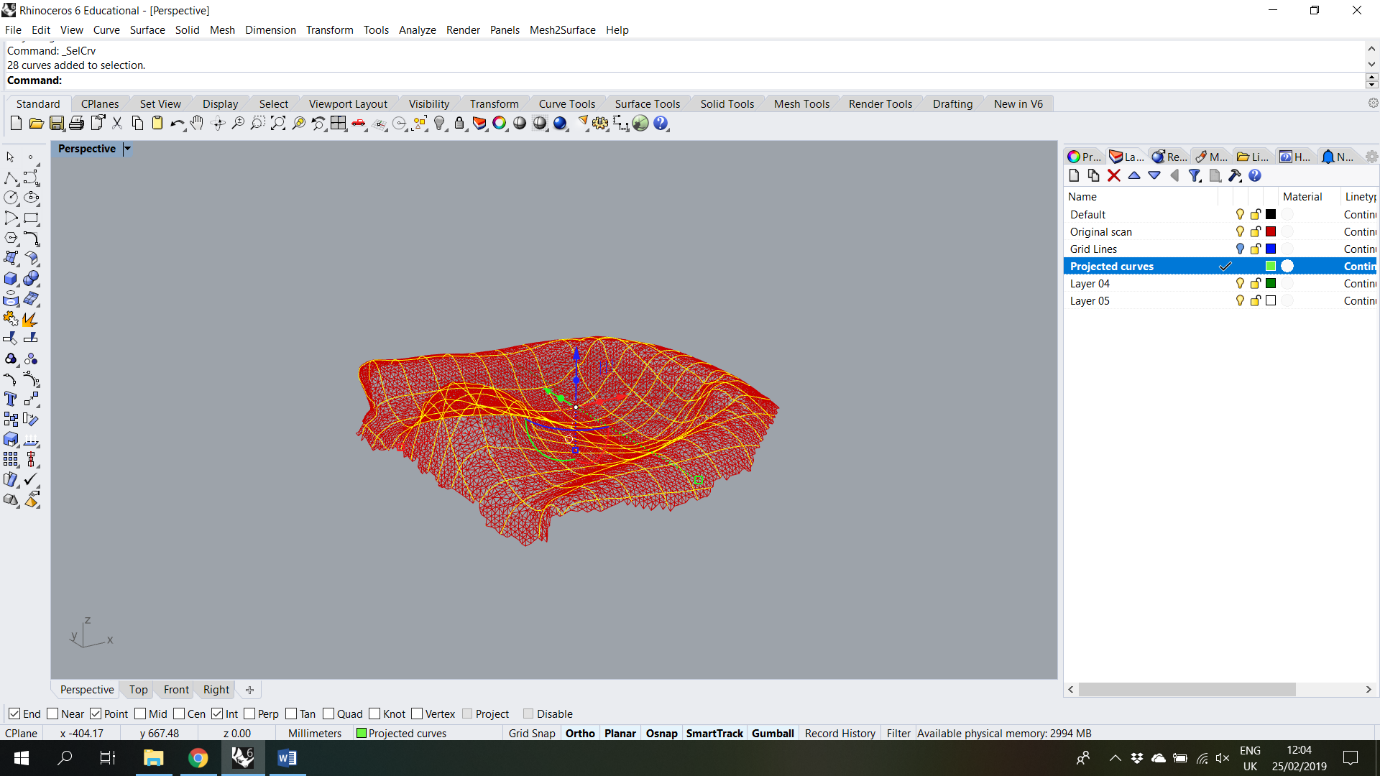


1. Edit → Rebuild. In the Rebuild pop-up window, lower the number of points to 30 or 40 points (can always change this later). Click Preview to see how the curve changes due to the change in the number of Control Points that sets the curves shape. Make sure the curves are Degree 3. Click OK. The curves are rebuilt with fewer control points.


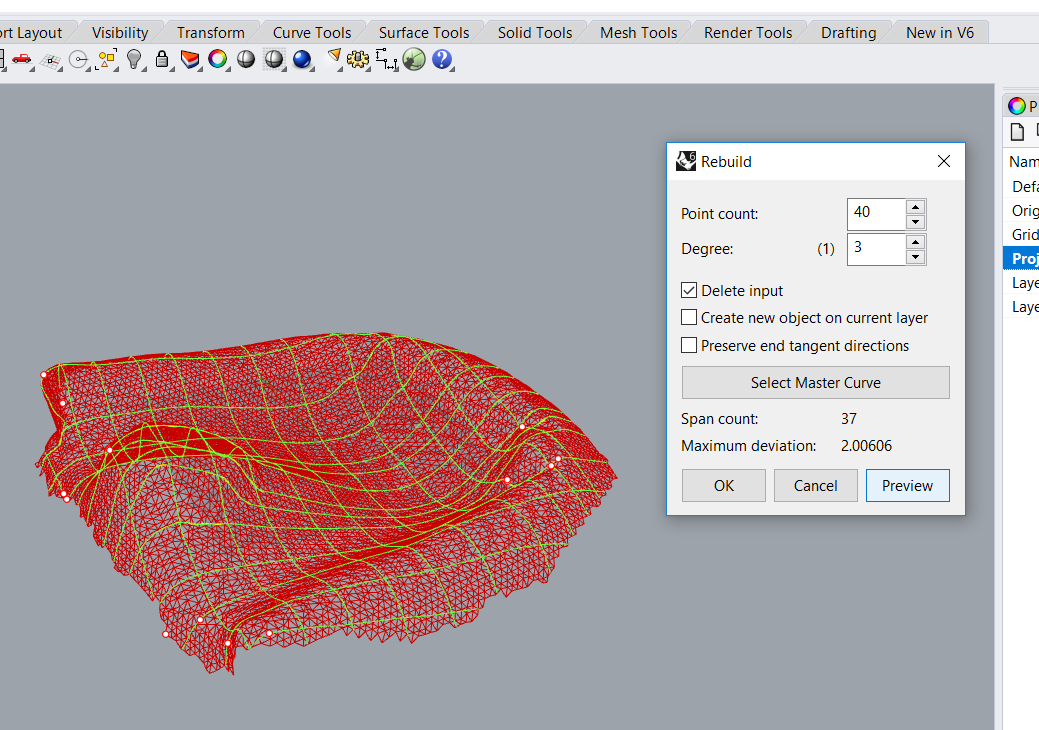


1. Turn the *Original scan* layer off.
2. Edit → Select objects → Curves. This will highlight all of the projected curves on the scan.
3. Use the command “Surface from network of curves”. The location of the command in Rhino is shown in the following figure.


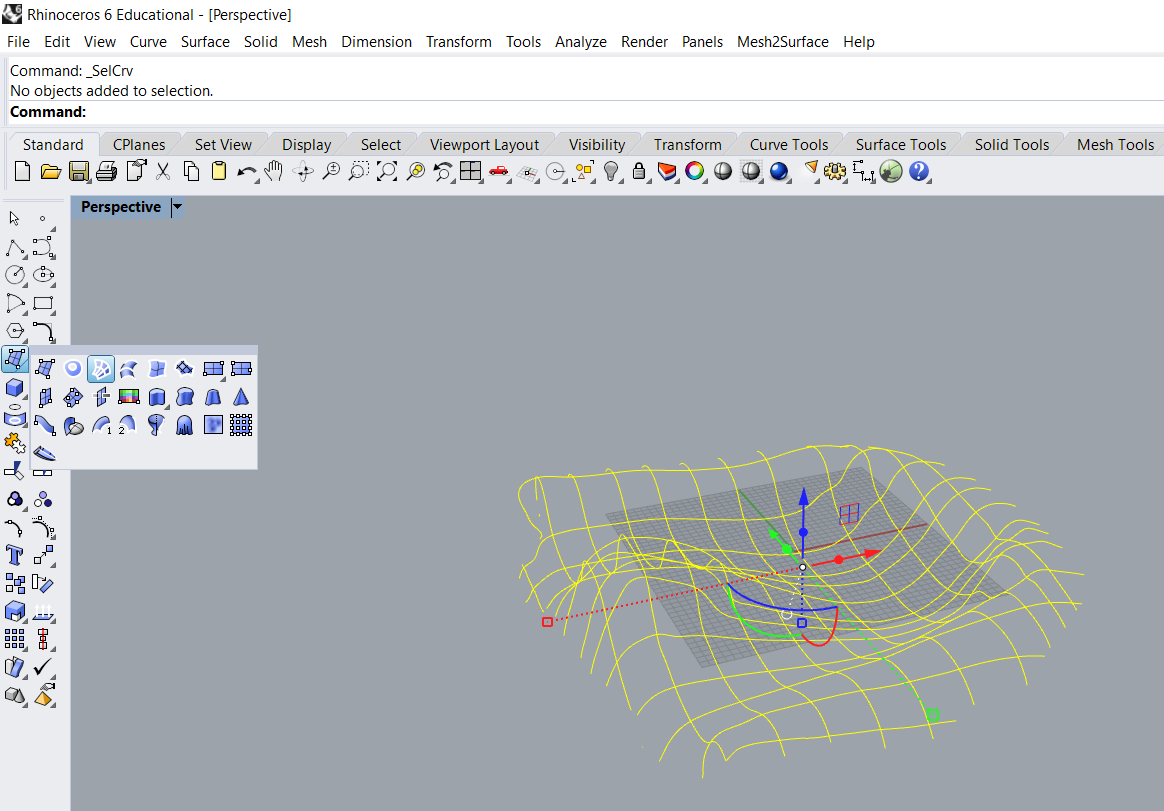


- 1. If needed, select one-by-one curves in one direction in order, press Enter, then repeat for the other direction of curves, and press Enter. A new surface should appear.

Set the number of Edge Curves and Interior curves as desired (5-10).
