# Supplementary material for: Development and evaluation of a facile mesh-to-surface tool for customised wheelchair cushions
Source: 3D Print Med. 2023 Feb 13;9:3. doi: 10.1186/s41205-022-00165-5 (PMC9926538; doi:10.1186/s41205-022-00165-5)

Additional file 2

Steps to turn a cushion scan file into an editable CAD surface:

1. Open Rhino3D software
2. Open the Grasshopper plug-in by clicking on Tools 🡪 Grasshopper. Grasshopper opens as a new window over Rhino.


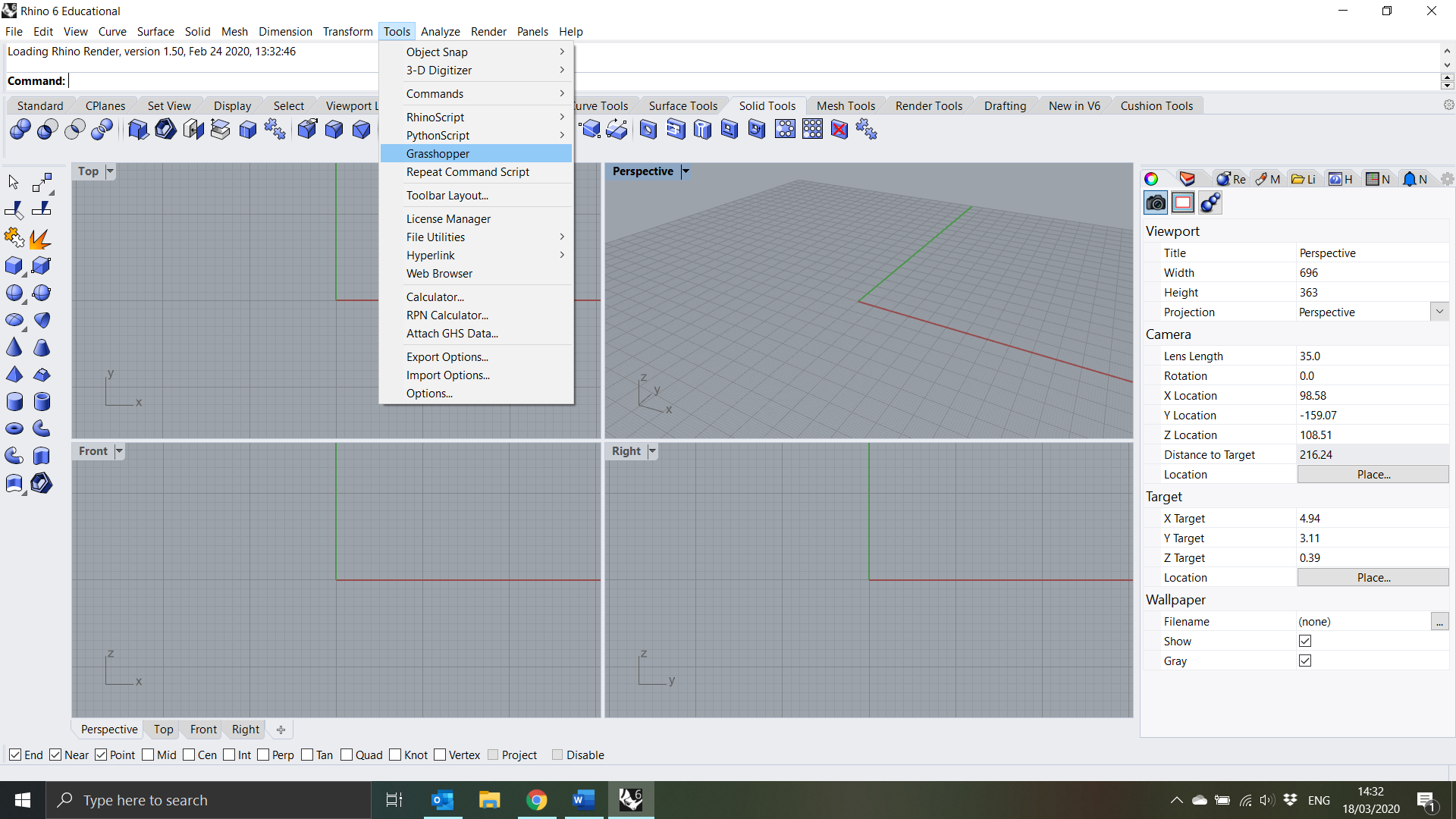


1. In Grasshopper (GH), go to File 🡪 Open Document… and find the file called “BackSupportCreation_UI.gh”. Click this file, then click Open. The screen may now look something like this:


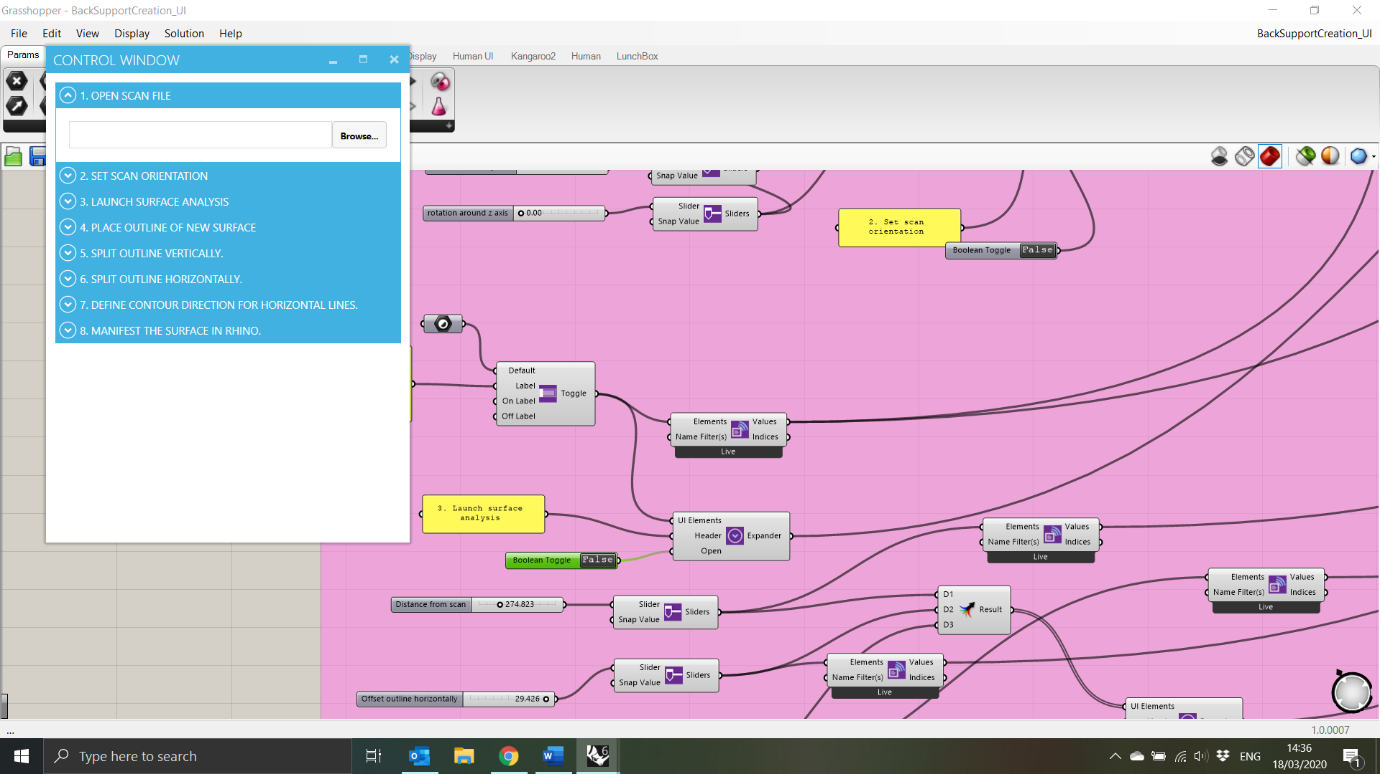


1. Move the Control Window to one side by clicking and dragging the window by its title tab. Then make GH smaller in the screen by clicking and dragging its corner when a double-ended arrow appears. Make it small enough so you can see the Rhino3D application. Your screen should now look something like the next picture, and enables you to use both GH and Rhino tools:


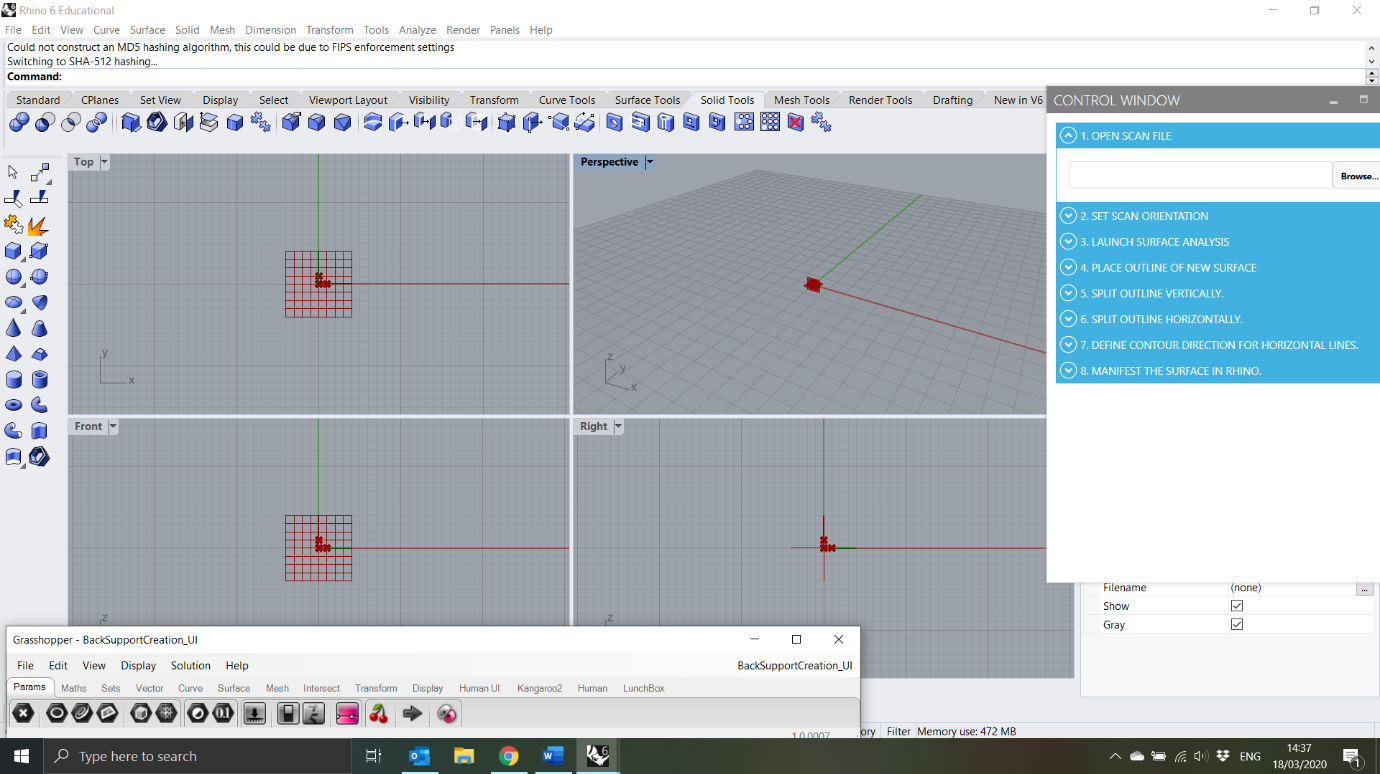


1. Next import the cushion/back support scan file (.stl). To do this, click the Browse button in the Control Window tab “1. OPEN SCAN FILE”. If you do not see the Browse button, click the down arrow to the left of “1. OPEN SCAN FILE”. A file browsing window will pop up, and you should select the scan file you want to work with, then click Open.

If the file has imported correctly, the scan model will appear in Rhino as a semi-transparent red object. To see the entire object, you may need to zoom out in each Viewport. The next picture shows an example of how you want your screen.


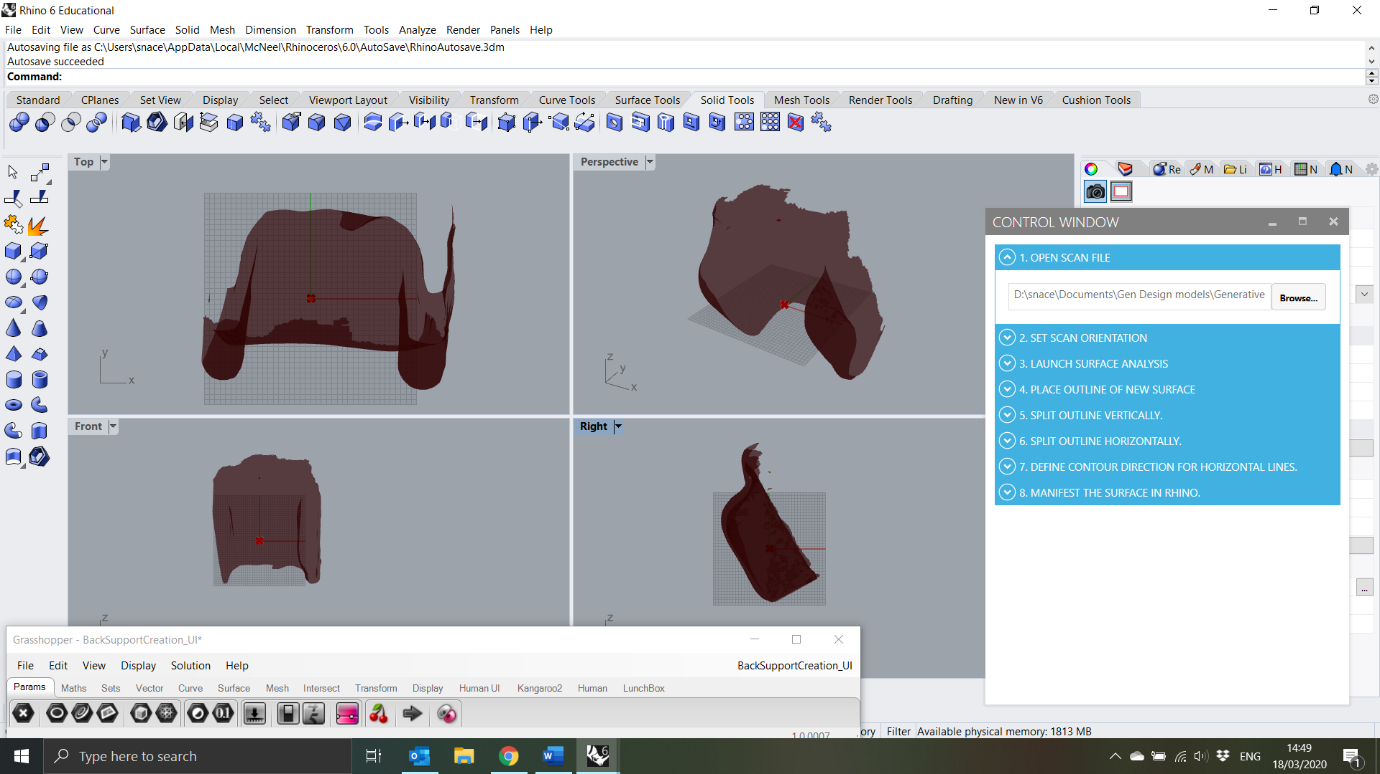


1. In the Control Window, open the next tab “2. SET SCAN ORIENTATION” by clicking on the down-arrow at the left corner of the tab.
   1. Using the “rotation around x-axis” slider first, then the proceeding sliders, orient the scan such that it looks similar to the back support in each of the Viewports below:


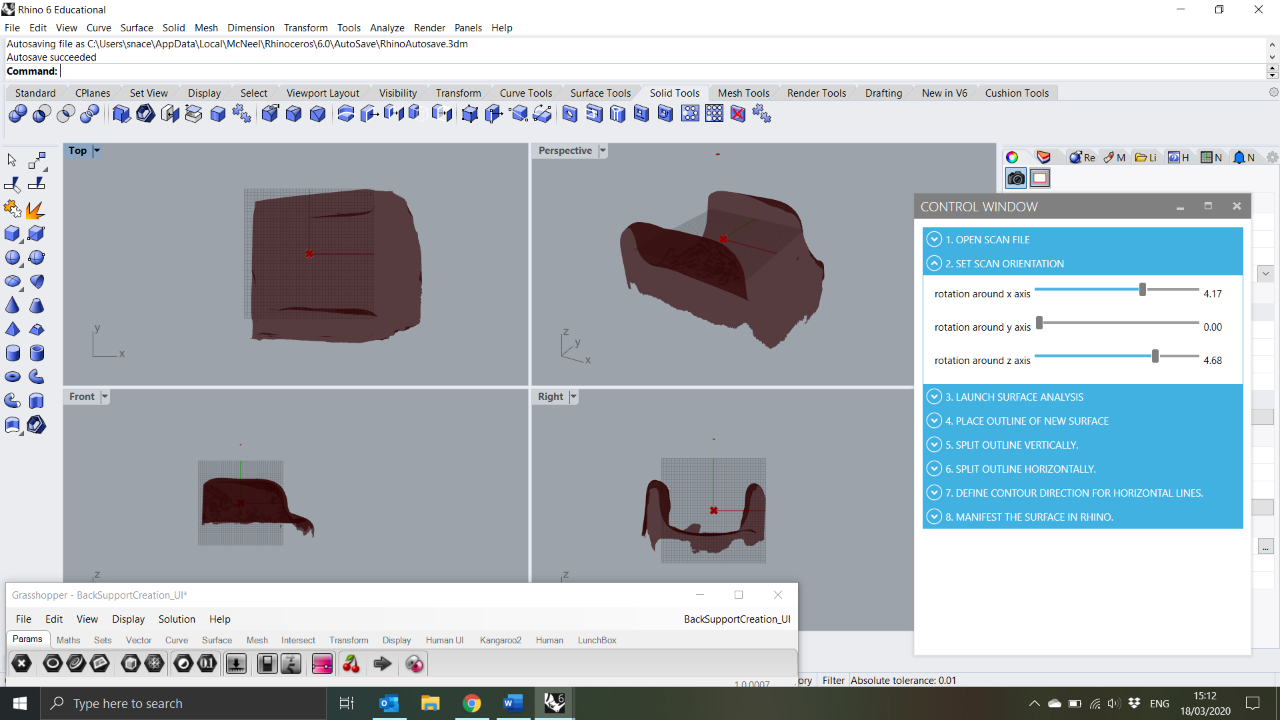


1. When finished orienting, open the third tab: “3. LAUNCH SURFACE ANALYSIS”. Switch the toggle to ON by clicking the switch **ONCE**. Wait until your screen looks something like the next picture before continuing to the next step:


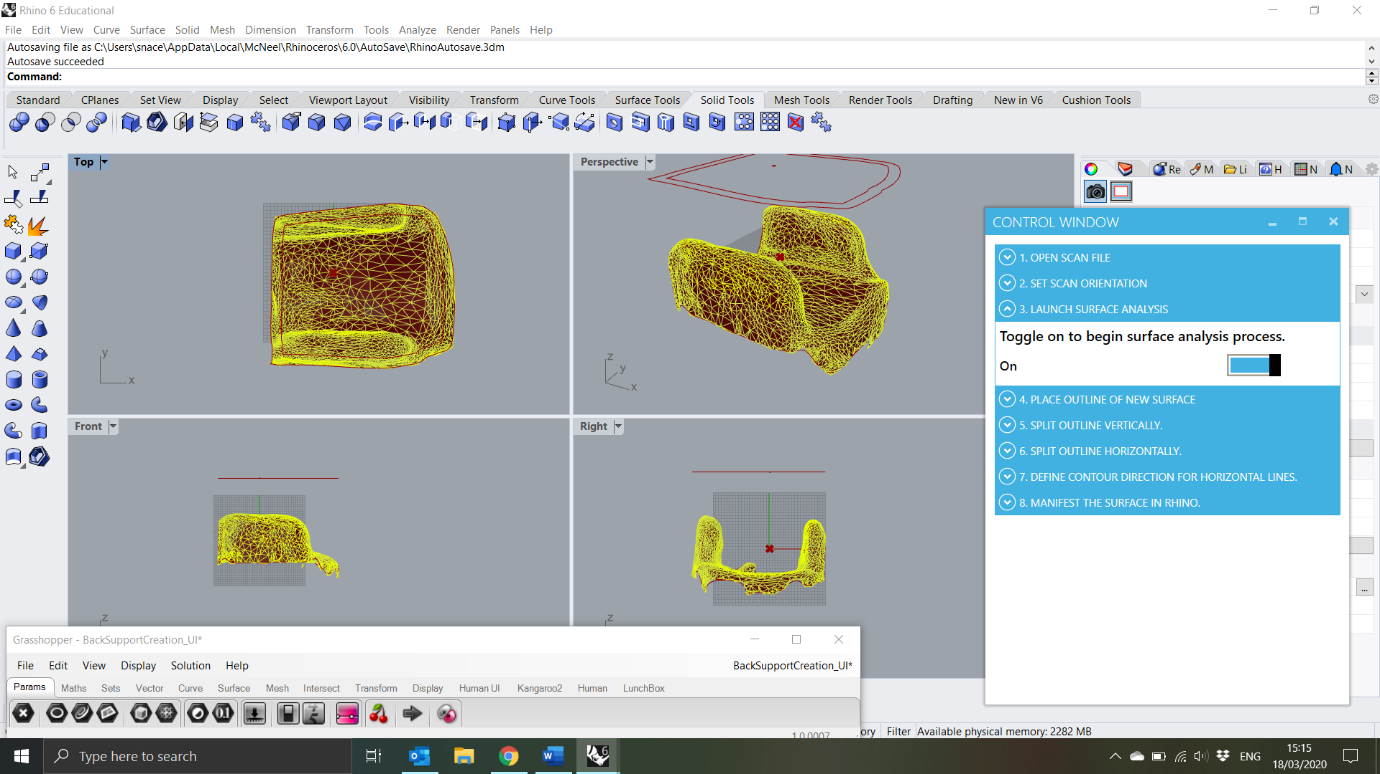


1. Open the next tab “4. PLACE OUTLINE OF NEW SURFACE”. If need be, use the first slider in the tab to move the red shape outlines closer to the scan object, so that you can see the red lines and the scan in each of the Viewports. Then, use the second slider to move the red outlines closer to each other, which will be easiest to see in the Top and Perspective Viewports. Once satisfied, click the toggle ONCE to place the outlines in Rhino before going to the next step.


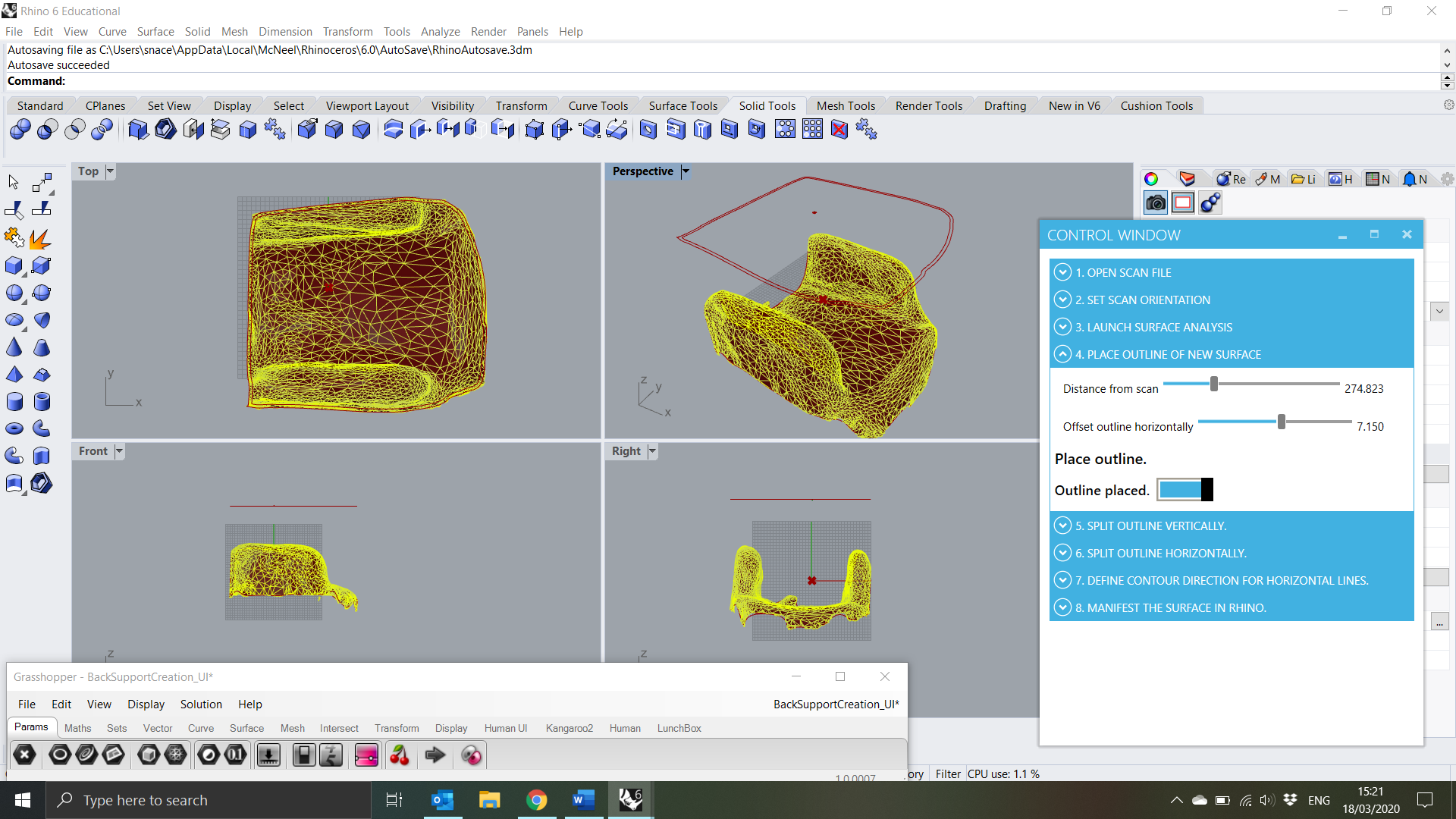


1. Open the next tab and follow the instructions in the tab.
   1. Once the “Vertical line” layer is the active layer, draw a line like that in the following picture using the Line command in the Rhino environment. Once it is drawn, align the vertical line with the red outlines; this is easiest to see in the Front Viewport, and you will need to zoom in to assure the lines are aligned.


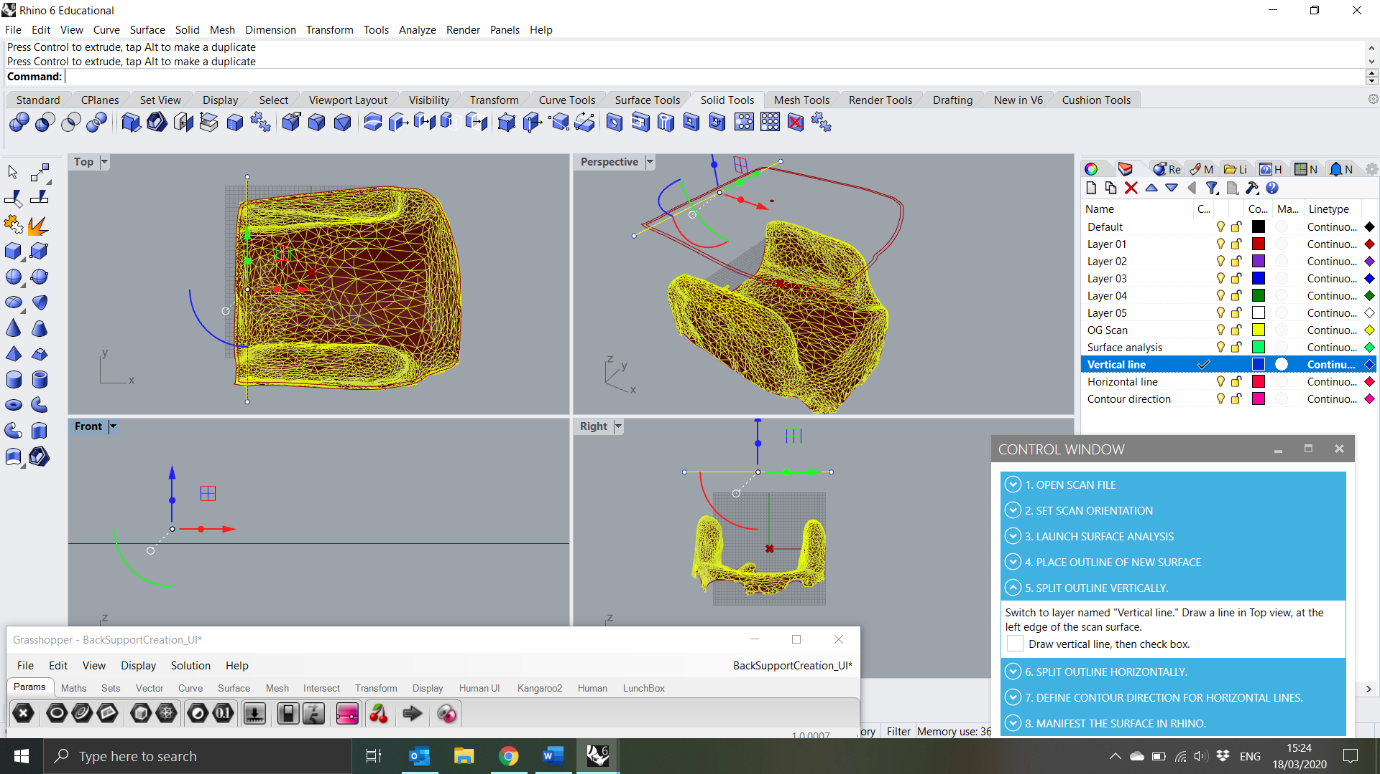


- 1. You will know the new line and the outline are aligned when X’s appear, like the following image. Check the box in the tab when done.


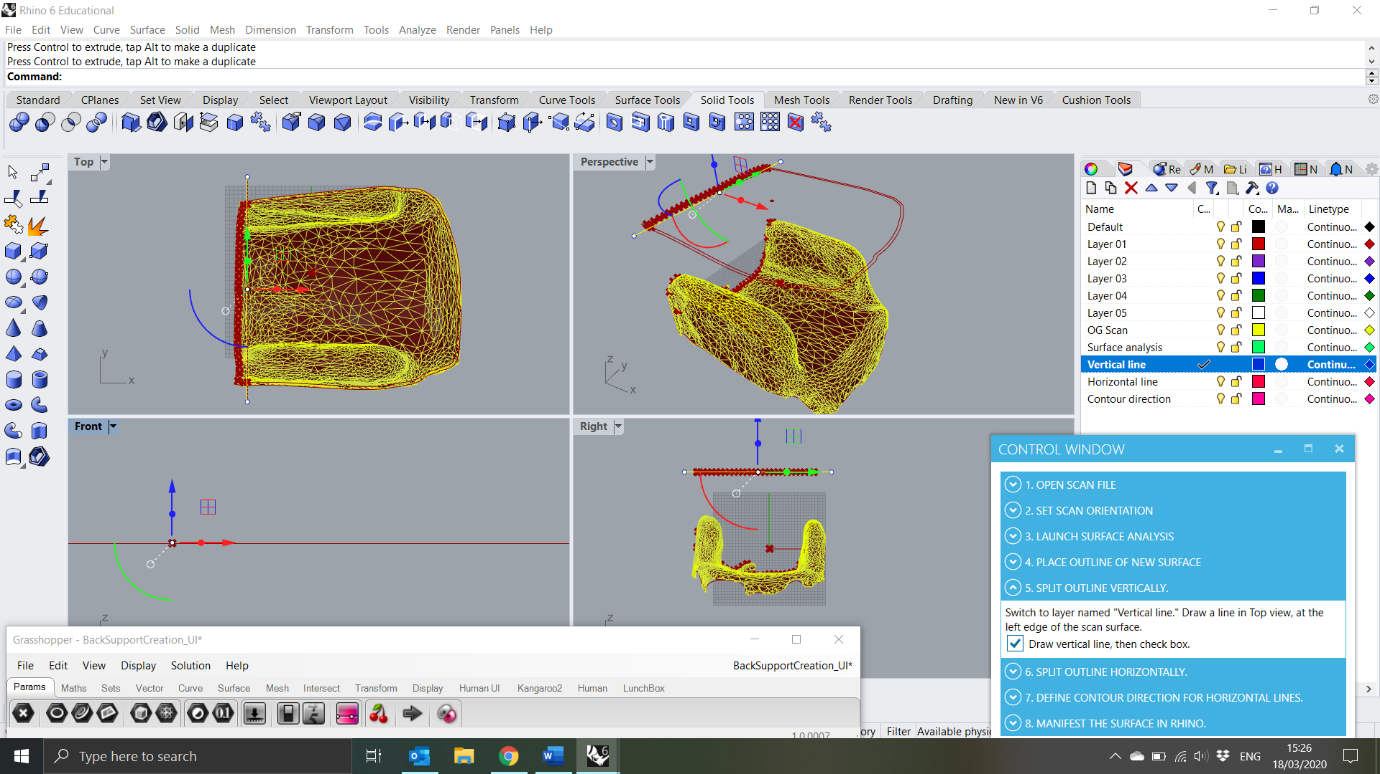


1. After checking the box in the last step, open the next tab: “6: SPLIT OUTLINE HORIZONTALLY”. Follow the directions in the new tab. Draw a horizontal line using similar steps as step 11, so that your screen looks like the next picture.


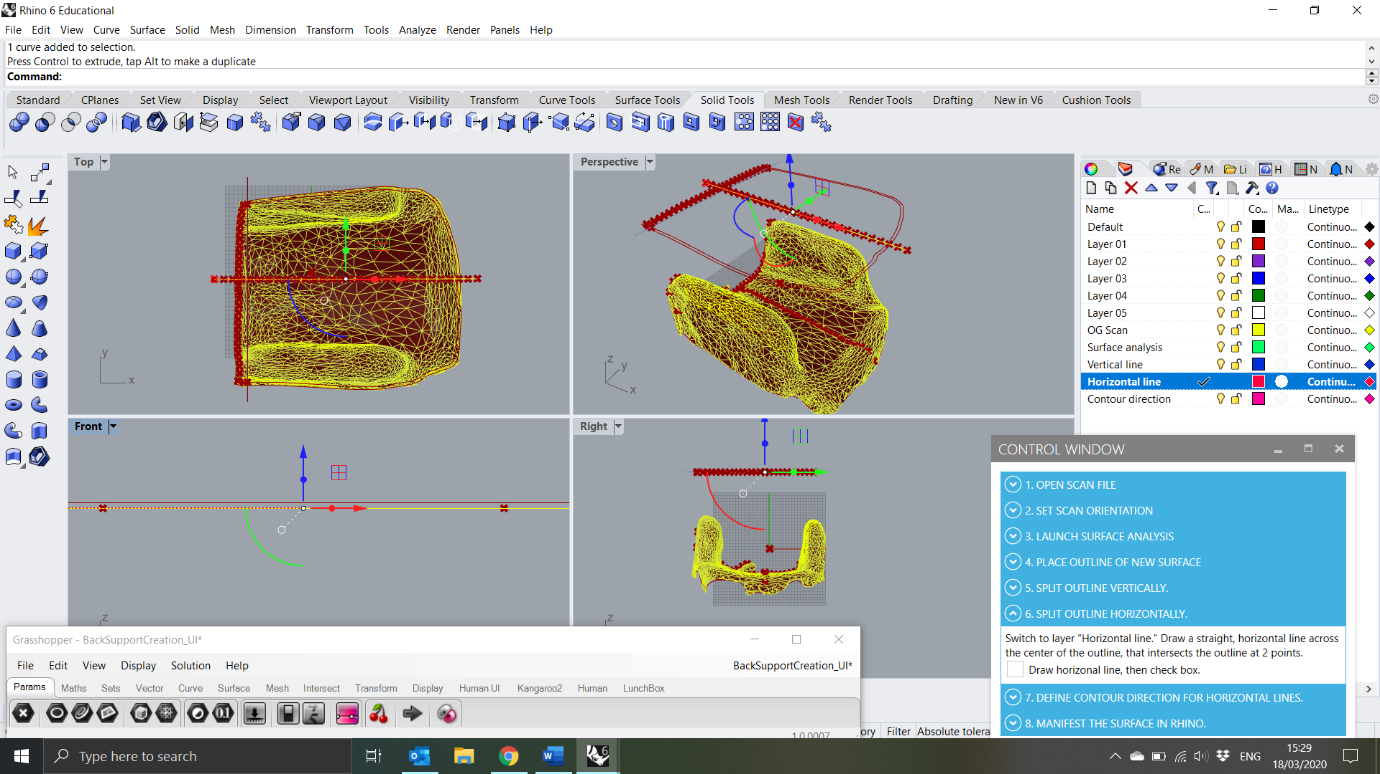


a. The horizontal line will need to be moved to align with the vertical line and the outline, just like before. You will know it is aligned when X’s appear all along the rest of your outline, as shown in the picture below. Check the box in the Control Window tab before moving to the next step.


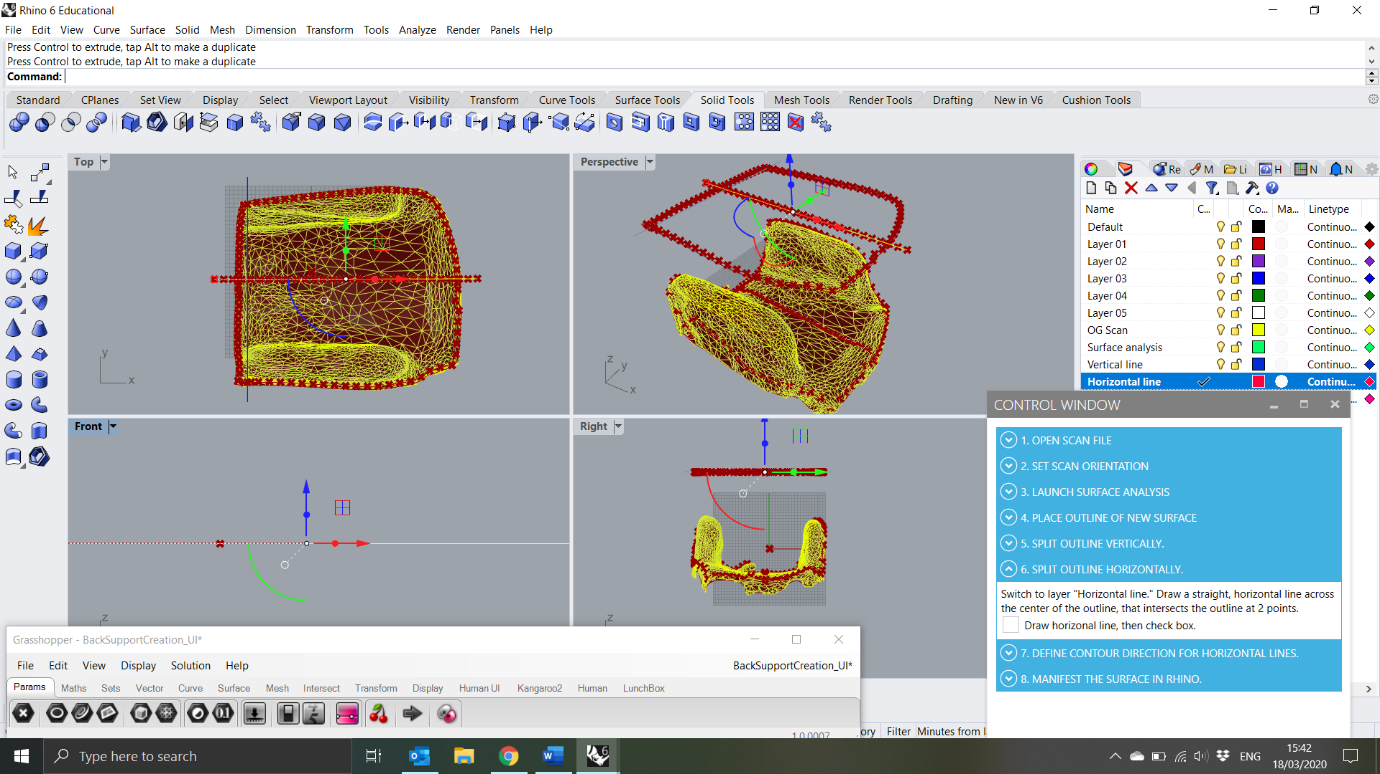


1. Once you have checked the box in the last step, open the next tab: “7. DEFINE CONTOUR DIRECTION FOR VERTICAL LINES”. Follow the directions written in the tab.
   1. Use Rhino’s Point command to draw a point. When placing the point in the Top Viewport, the following picture offers guidance on point placement. The pink pointer is the Point location.


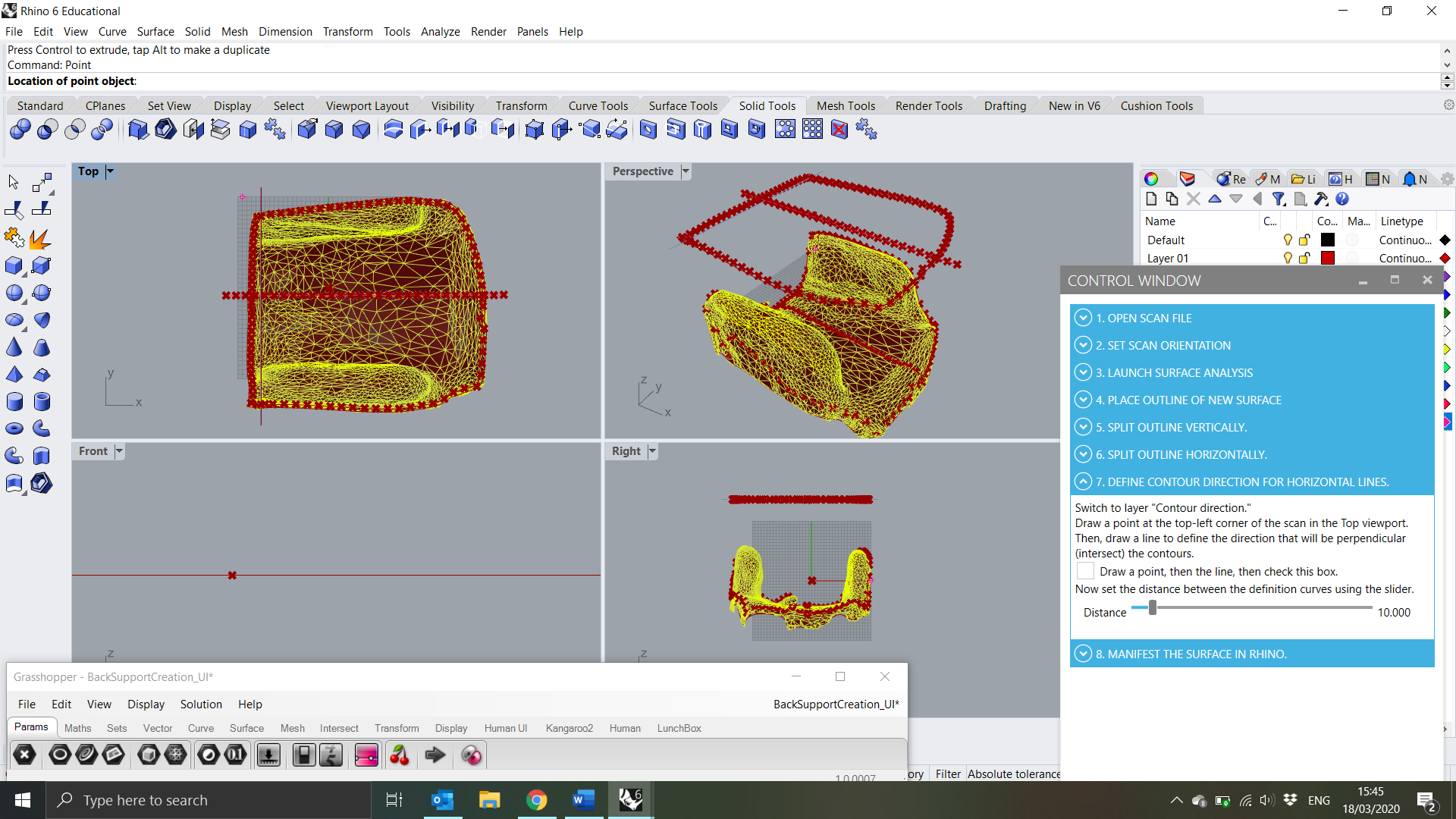


- 1. Draw a line using the Line command, draw a horizontal line that starts at your point and ends past the edge of your scan, like the picture below. The pink line is the new line. Check the box when you’ve drawn your line.


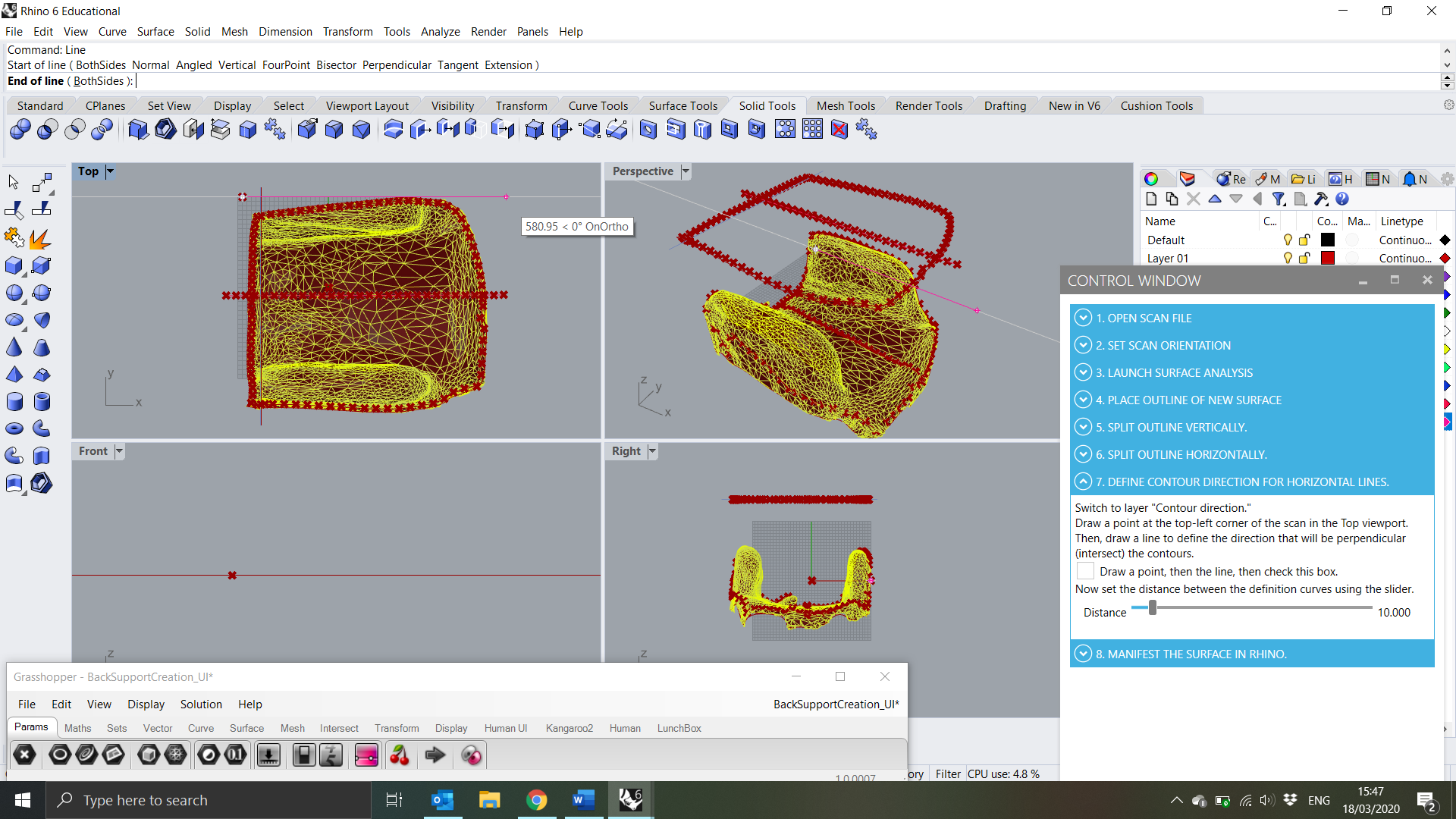


- 1. Using the slider, set the distance between contour lines. Sometimes, a distance will not be able to produce a new surface, so watch your scan as you move the slider. When a new surface is visible in the Viewport, you will know that distance is acceptable for making a new CAD-editable surface from the scan. The picture below shows an example.


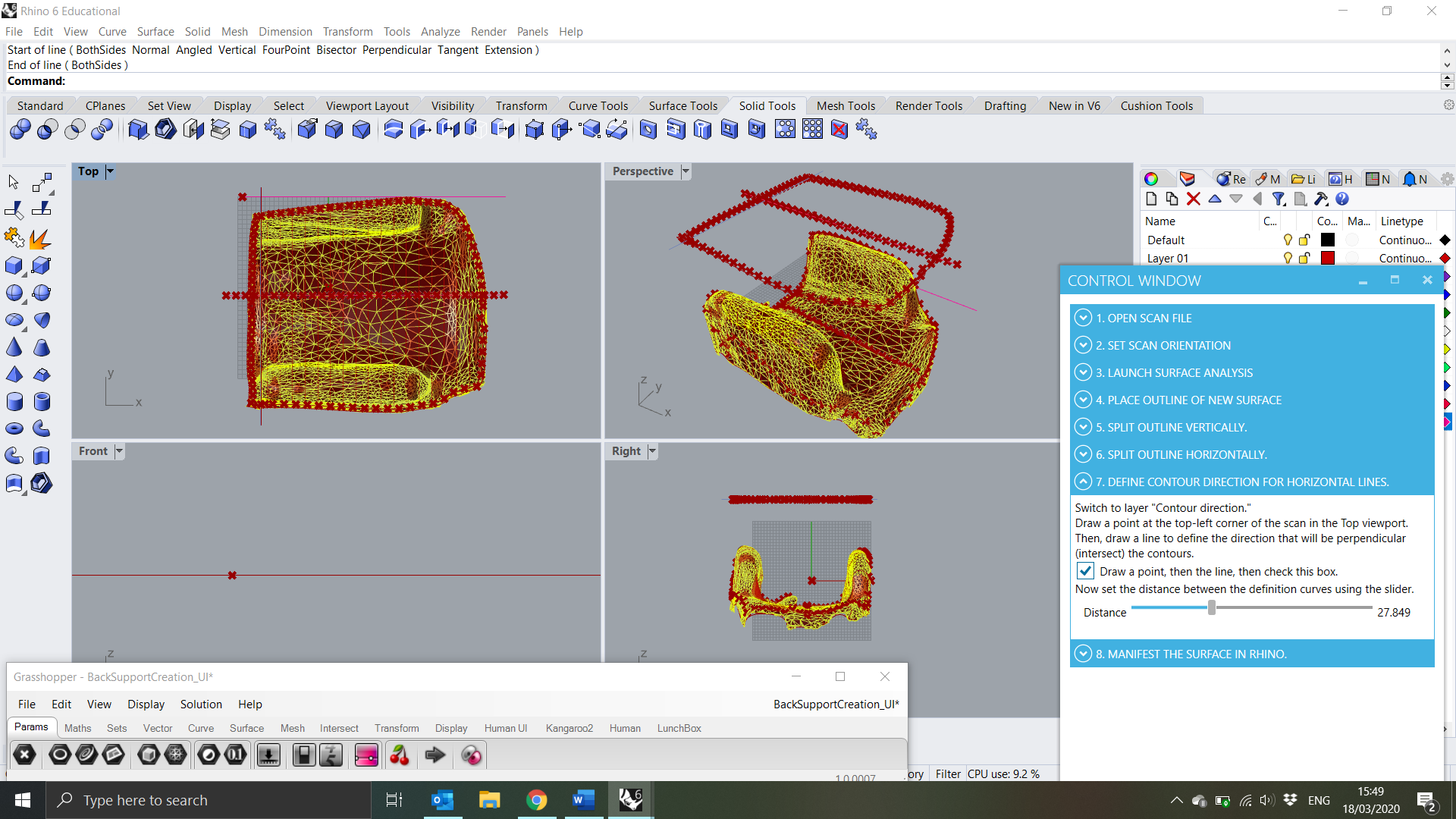


1. Open the last tab in the Control Window: “8. MANIFEST THE SURFACE IN RHINO”. Toggle the switch ONCE so that the tab reads BAKED. You will see your new surface in the Rhino environment, the white object in the next screenshot:


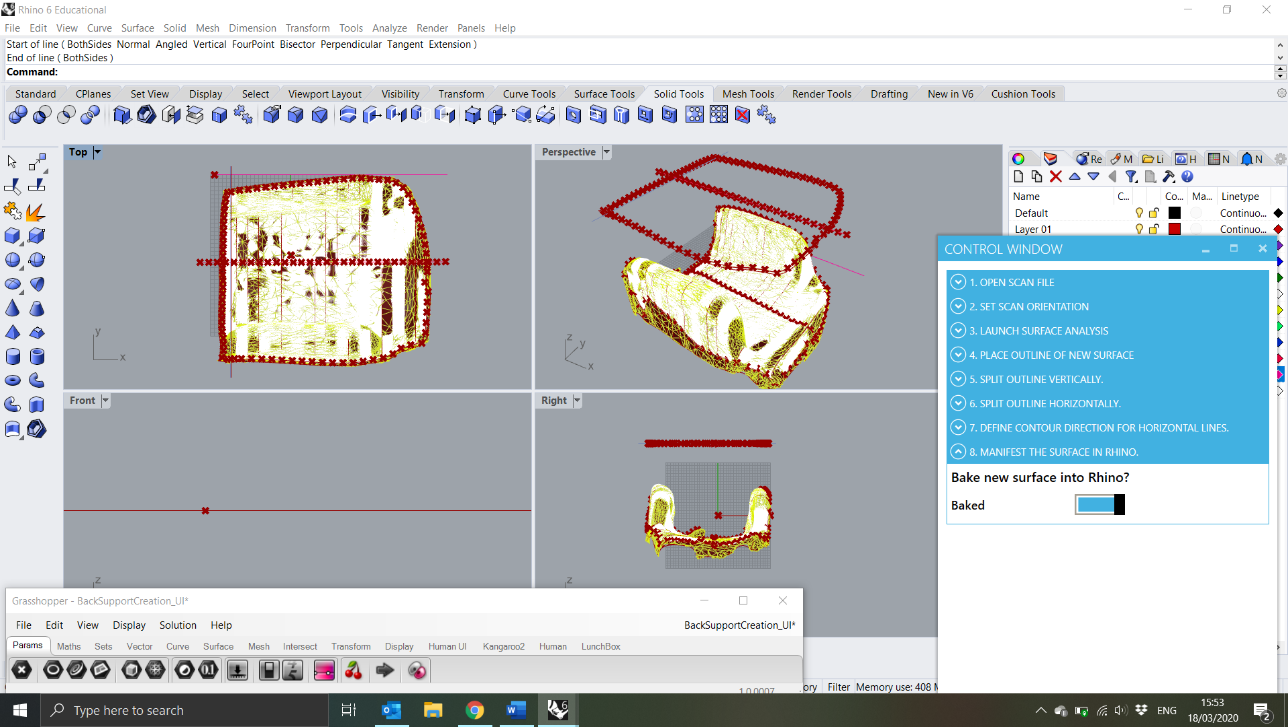

Supplement: Supplementary file 2 — Additional file 2. [file 41205_2022_165_MOESM2_ESM.docx]
