# Supplementary material for: Development and evaluation of a facile mesh-to-surface tool for customised wheelchair cushions
Source: 3D Print Med. 2023 Feb 13;9:3. doi: 10.1186/s41205-022-00165-5 (PMC9926538; doi:10.1186/s41205-022-00165-5)
Supplement: Supplementary file 3 — Additional file 3. [file 41205_2022_165_MOESM3_ESM.docx]

Additional file 3

## Survey questions for clinical user needs identification

- On a scale of 1-10, 1 being the easiest and 10 being the hardest: how was the tutorial?
- Did you have any difficulties or limitations during the tutorial? (software, purpose of scan, laptop use, understanding the tutorial?)
- How does this tutorial compare to the previous tutorial or to the previous process used to manipulate the scan? (manually doing the gridlines vs automated version?)
  - Has the process improved or not? If so, in what ways?
- How would you rate the ease of use? (locating the buttons or tools)
- Was there anything in the tutorial that you did not understand? Was there anything unclear?
- Would you recommend the software?
- How long do you think the tutorial should take? What is your opinion on the time it takes to finish the tutorial?
- What changes would you recommend:
  - To the software?
  - To the tutorial?
- What specific feature or tool did you find the most beneficial, if any?
